# Adverse events and impact on quality of life of antibody‐drug conjugates in the treatment of metastatic breast cancer: A systematic review and meta‐analysis

**DOI:** 10.1111/eci.70001

**Published:** 2025-02-13

**Authors:** Marta Perachino, Eva Blondeaux, Chiara Molinelli, Tommaso Ruelle, Irene Giannubilo, Luca Arecco, Simone Nardin, Maria Grazia Razeti, Roberto Borea, Diletta Favero, Chiara Lanzavecchia, Edoardo Chiappe, Loredana Tomasello, Elene Mariamidze, Kristina Jankovic, Mihaela Stana, Silvia Ottonello, Graziana Scavone, Luciana de Moura Leite, Stefano Spinaci, Cristina Saura, Matteo Lambertini

**Affiliations:** ^1^ U.O. Clinica di Oncologia Medica IRCCS Ospedale Policlinico San Martino Genoa Italy; ^2^ Department of Internal Medicine and Medical Specialties (DiMI), School of Medicine University of Genova Genoa Italy; ^3^ Medical Oncology Service Vall d'Hebron University Hospital and Vall d'Hebron Institute of Oncology Barcelona Spain; ^4^ U. O. Epidemiologia Clinica IRCCS Ospedale Policlinico San Martino Genoa Italy; ^5^ Section of Medical Oncology, Department of Precision Medicine in Medical, Surgical and Clinical Care (Me.Pre.C.C) University of Palermo Palermo Italy; ^6^ Oncology and Hematology Department Todua Clinic Tbilisi Georgia; ^7^ Clinic of Oncology University Clinical Center Nis Nis Serbia; ^8^ Medical Oncology Department “Elysee Hospital” Alba Iulia Romania; ^9^ Department of Experimental Medicine (DIMES) University of Genova Genoa Italy; ^10^ Department of Medical Oncology A.C. Camargo Cancer Center São Paulo Brazil; ^11^ Department of Surgery Ospedale di Villa Scassi ASL 3 Genoa Italy

**Keywords:** adverse events, antibody‐drug conjugate, breast cancer, quality of life, toxicity

## Abstract

**Background:**

Antibody‐drug conjugates are novel effective therapies for metastatic breast cancer. Nevertheless, their toxicity profile can significantly affect patients' quality of life over time.

**Methods:**

This is a systematic review and meta‐analysis of randomized controlled trials of antibody‐drug conjugates currently approved for the treatment of metastatic breast cancer [trastuzumab‐emtansine (T‐DM1), trastuzumab deruxtecan (T‐DXd) and sacituzumab‐govitecan (SG)] versus standard therapy to evaluate the risk of adverse events, discontinuation rate due to toxicity, impact on quality of life according to EORTC QLQ‐C30 scale and subdomains. Relative risks (RR) and hazard ratios (HR) with 95% CIs were calculated using random effects models.

**Results:**

Nine trials with a total of 5753 patients were included. The most common adverse events of any grade for T‐DM1 included thrombocytopenia (RR 7.14, 95% CI 4.13–12.36) and increased alanine‐transaminase (ALT) (RR 2.04, 95% CI 1.43–2.91), for T‐DXd were nausea (RR 2.39, 95% CI 1.90–3.00) and anemia (RR 1.55, 95% CI 1.27–1.90), while for SG were neutropenia (RR 1.30, 95% CI 1.14–1.49), diarrhea (RR 3.62, 95% CI 2.97–4.42) and nausea (RR1.90, 95% CI 1.65–2.19). Severe adverse events such as interstitial lung disease and left ventricular dysfunction were peculiar of T‐DXd. Antibody‐drug conjugates significantly delayed clinical deterioration of global health status by EORTC QLQ‐C30 (HR .71, 95% CI .59–.86), physical, emotional and social functioning, pain and fatigue symptoms.

**Conclusions:**

This meta‐analysis offers consolidated data on adverse events associated with antibody‐drug conjugates and their effects on patients' quality of life, emphasizing differences based on the specific agent. These findings underscore the critical need for effective strategies to prevent, diagnose and manage these toxicities.

## Graphical abstract

This systematic review and meta‐analysis examined RCTs comparing antibody‐drug conjugates (T‐DM1, T‐DXd and SG) with standard therapies for metastatic breast cancer. It assessed adverse events, treatment discontinuation due to toxicity and quality of life using the EORTC QLQ‐C30 scale. The findings highlight distinct adverse event profiles across ADCs and their impact on patients' quality of life highlighting the importance of strategies to mitigate these toxicities for improved patient outcomes.

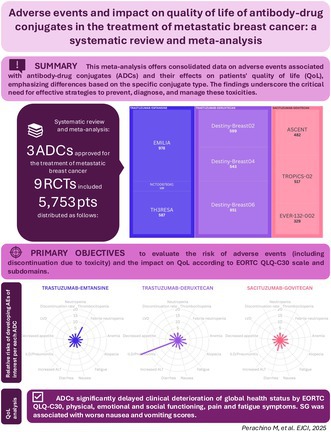

## 1 INTRODUCTION

Antibody‐drug conjugates (ADCs) have revolutionized the treatment landscape of numerous tumours and their implementation in the treatment algorithm of metastatic breast cancer is in continuous expansion. In the last years, different molecules have been approved by the Food and Drug Administration (FDA) and the European Medicine Agency (EMA), expanding the therapeutic armamentarium, and improving the survival of patients.

Trastuzumab‐emtansine (T‐DM1), a HER2‐targeting drug, was the first approved ADC by the FDA in 2013, after the results of the EMILIA trial, which demonstrated a progression‐free survival (PFS)^1^ and overall survival (OS)^2^ improvement in patients with advanced HER2‐positive breast cancer pretreated with trastuzumab and a taxane. Later, this drug received also approval in the adjuvant setting for patients with residual disease after neoadjuvant trastuzumab plus chemotherapy.^3^

The second ADC approved was trastuzumab‐deruxtecan (T‐DXd) in HER2‐positive metastatic breast cancer, pretreated with at least one HER2‐targeted regimen in the metastatic setting. This was after the exciting results of Destiny‐Breast01,^4^ with its efficacy confirmed by Destiny‐Breast02^5^ and Destiny‐Breast03,^6^ being the current standard second‐line therapy for these patients.

Recently, T‐DXd indication was expanded also to HER2‐low disease, defined as Immunohistochemistry (IHC) 1+ or IHC 2+/In Situ Hybridization (ISH)‐negative, with some evidence of its potential efficacy also in HER2 ultralow disease (IHC 0 with membrane staining)^7^. Several trials are ongoing with the potential to expand T‐DXd use in first‐line (Destiny‐Breast09 NCT04784715) and in the neoadjuvant (Destiny‐Breast11 NCT05113251) or adjuvant (Destiny‐Breast05 NCT04622319) settings.

Sacituzumab govitecan (SG), a TROP2‐targeting ADC, has shown to improve PFS and OS in pre‐treated patients with metastatic triple‐negative^8^ and hormone receptor‐positive/HER2‐negative^9^ disease. SG is currently being studied as first‐line therapy (ASCENT‐07 NCT05840211) and in the adjuvant setting (ASCENT‐05 NCT05633654).

Despite being engineered with high selectivity for their target in order to limit toxicities, ADCs can lead to different treatment‐related adverse events (AEs), which could be as high as 91.2% considering all grade toxicities and 46.1% when looking at grade ≥3, with a discontinuation rate of 13.2%.^10^

Intrinsically to their structure, each component of the molecule of an ADC can contribute to their peculiar toxicity, depending on the normal tissue expression of the target antigen (‘on‐target toxicity’) or on the cytotoxic payload, including when it is released in the bloodstream (‘off‐target toxicity’). Moreover, it may cause ‘on‐tumour’ toxicity when the payload is released in the tumour microenvironment in the absence of a target as well as ‘off‐tumour’ toxicity when it causes toxicity by its release in normal tissue.^11^

The safety profile of the ADCs was commonly reported in the registration trials that led to their approval; ADC‐induced short‐ and long‐term toxicities could affect patients' quality of life (QoL) and should be weighed in the balance with the effectiveness profile, being of strategic importance in the decision‐making process. For this purpose, patient‐reported outcomes (PROs) and health‐related quality of life (HRQoL) questionnaires can serve as tools designed to gauge patients' experiences with anticancer treatments and enhance the physician's capacity to evaluate the impact of the drug beyond its efficacy profile.

To date, there are no head‐to‐head comparisons of the safety spectrum of ADCs for metastatic breast cancer considering PROs nor their impact on discontinuation rate or QoL. To address these questions, we performed a systematic review and meta‐analysis of the randomized controlled trials (RCTs) that compared the ADCs currently approved by the FDA/EMA for the treatment of metastatic breast cancer to standard chemotherapy concerning the rate of AEs, discontinuation rate due to toxicity and impact on QoL.

## 2 METHODS

### 2.1 Search strategy, study selection and data extraction

Preferred Reporting Items for Systematic Reviews and meta‐Analyses guidelines were used for the conduct and reporting of this systematic review.^12^ The meta‐analysis protocol was registered in the PROSPERO database under the following registration number: CRD 42024511732.

RCTs including patients with unresectable or metastatic breast cancer, irrespective of the biological subtype, were included if they compared ADC versus standard treatment arm (chemotherapy ± target therapy for HER2‐positive tumours). We decided to include only RCTs that evaluated ADCs that have already received the FDA/EMA approval (T‐DM1, T‐DXd and SG).

Clinical trials were identified by a computerized search through the PubMed search database with the string (‘antibody‐drug conjugate’[Title/Abstract] OR ‘antibody‐drug conjugates’[Title/Abstract] OR ‘ADC’[Title/Abstract] OR ‘ADCs’[Title/Abstract]) AND (((‘randomized controlled trial’[Publication Type] OR ‘controlled clinical trial’[Publication Type] OR ‘randomized’[Title/Abstract] OR ‘clinical trials as topic’[MeSH Terms] OR ‘randomly’[Title/Abstract] OR ‘trial’[Title]) NOT (‘animals’[MeSH Terms] NOT ‘humans’[MeSH Terms])) AND (‘breast neoplasms’[MeSH Terms] AND (‘advance’[Title/Abstract] OR ‘advanced’[Title/Abstract] OR ‘metastatic’[Title/Abstract] OR ‘metast*’[Title/Abstract] OR (‘unresectability’[Title/Abstract] OR ‘unresectable’[Title/Abstract] OR ‘unresected’[Title/Abstract])))).

The search was performed with no date restriction up to March 4, 2024.

In the analysis, we included also abstracts and conference papers from the annual meetings of the European Society for Medical Oncology (ESMO) and the American Society of Clinical Oncology (ASCO) as well as from the San Antonio Breast Cancer Symposium (SABCS). Supplementary material was also reviewed when available.

The following information was extracted from each report: name of study/study code, first author and year of publication, journal, study design, treatment arms, number of patients enrolled in the study, number of patients evaluated for the safety analysis, rate of the key AEs, DR due to toxicity.

The first outcome of interest in this meta‐analysis was the relative risk (RR) of AEs. The incidence and severity of the following key AEs were collected: neutropenia, thrombocytopenia, febrile neutropenia, anemia, alopecia, fatigue, nausea, diarrhea, interstitial lung disease (ILD)/pneumonitis, increased alanine‐transaminase (ALT), decreased appetite and left ventricle dysfunction (LVD). In each trial, the severity was evaluated according to Common Terminology Criteria for Adverse Events (CTCAE) grade, with further categorization as both ‘all grade’ (CTCAE G1–G5) and as ‘severe grade’ (≥G3) AEs for each endpoint. We reported the rate of each AE collected from the most recent publication.

### 2.2 HR‐QoL assessments

For this analysis, additional information was retrieved from the included RCTs: number of patients evaluated for PROs, available PROs assessed using the European Organization for Research and Treatment of Cancer Quality of Life of Cancer Patients (EORTC QLQ‐C30) questionnaire, availability of HRs of global health status (GHS)/QoL domain, physical functioning, emotional functioning, social functioning, pain symptoms, fatigue symptoms, nausea and vomiting symptoms.^13^

The endpoint considered for these outcomes of interest was time from baseline to first deterioration (TTD) in PROs, defined as the time from baseline to the first clinically significant deterioration in PROs.

### 2.3 Data analysis

RRs with 95% CI estimation were extracted from included studies or derived from the number of events of each arm computed as the ratio of proportions of events between groups. When RRs and HRs were not available or could not be computed for a specific outcome, the studies were excluded from that specific analysis. Pooled RRs, and HRs with their 95% CI were calculated using the method of DerSimonian and Laird using the random effects model.^14^ The quantitative measure of the degree of inconsistency in the results of the included studies was computed using the Higgins *I* ^2^ index.^15^ Pooled RRs and HRs were considered statistically significant with a *p* value of .05 (two‐sided).

Data were independently extracted by two investigators (M.P. and C.M.) to ensure homogeneity of collection and to rule out the effect of subjectivity in data gathering and entry. Disagreements were resolved by iteration, discussion and consensus with a third author (M.L.).

## 3 RESULTS

**FIGURE 1:**
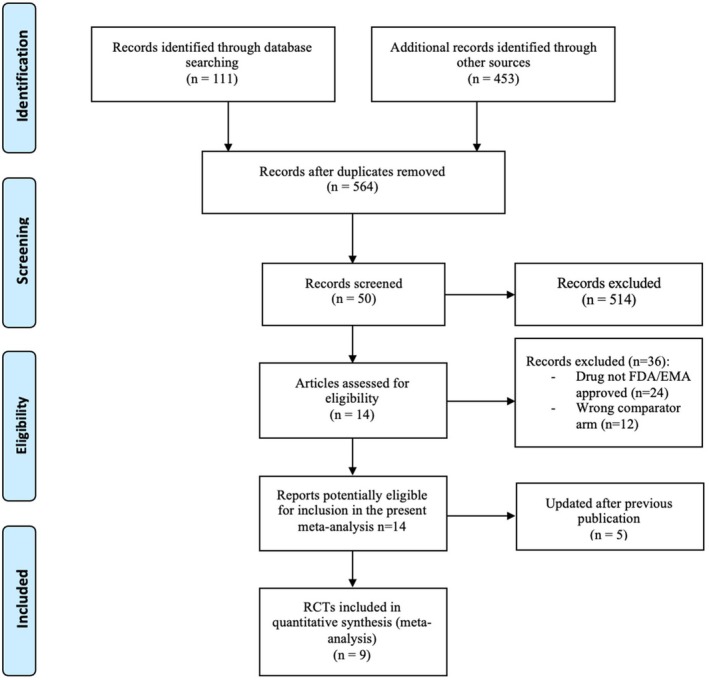
Prisma flow diagram depicting the search strategy in the systemic review literature search.

Of the 49 identified records, nine RCTs were included in the present meta‐analysis (Figure 1).^1^, ^2^, ^5^, ^7^, ^8^, ^16^, ^17^, ^18^, ^19^, ^20^, ^21^, ^22^, ^23^, ^24^, ^25^ A total of 5753 patients were randomized in these RCTs, of whom 3227 were allocated to the ADC arm and 2526 to the standard therapy arm. Three trials evaluated T‐DM1, three T‐DXd and three SG versus standard treatment. All of them were phase III trials except for one that was a randomized phase II trial.^20^ For all the trials involving T‐DM1, the population enrolled included HER2‐positive metastatic breast cancer, while for T‐DXd one trial accrued patients with HER2‐positive metastatic disease while two trials HER2‐low/ultralow metastatic breast cancer. In two trials, SG was evaluated in a population of patients with hormone receptor‐positive breast cancer and in one trial in those with triple‐negative disease. The characteristics of the studies included are summarized in Table 1.

**TABLE 1:** RCTs included in the meta‐analysis.

Trial name	First author, year of publication	Phase	Masking	Enrolled population	Treatment arms	Median age	Number of patients enrolled	Number of patients evaluated for AEs	Number of patients evaluated for PROs
EMILIA	S. Verma, N Eng J Med, 2012 V. Dieras, Lancet Oncol 2017	III	Open‐label	HER2+ mBC previously treated with trastuzumab and a taxane	T‐DM1 (3.6 mg/kg, IV q3w)	53 (25–84)	495	490	–
					Capecitabine (orally 2000 mg/m^2^) + Lapatinib (orally 1250 mg 1‐21q3w)	53 (24–83)	496	488	–
NCT00679341	S.Hurvitz, J Clin Oncol 2013	II	Open‐label	HER2+ mBC without prior chemotherapy or trastuzumab for metastatic disease	T‐DM1 (3.6 mg/kg, IV q3w)	55 (27–82)	67	69*	–
					Trastuzumab (8 mg/kg loading dose followed by 6 mg/kg, IV q3w) + Docetaxel (75 or 100 mg/m2, IV q3w)	52 (33–75)	70	66	–
TH3RESA	I. Krop, Lancet Oncol 2014 I. Krop, Lancet Oncol 2017	III	Open‐label	HER2+ mBC previously treated with both trastuzumab and lapatinib (advanced setting) and a taxane (any setting) and with progression on two or more HER2‐directed regimens in the advanced setting	T‐DM1 (3.6 mg/kg, IV q3w)	53 (27–89)	404	403	297
					CT (any single agent), HT for ER+ (single agent or dual therapy), anti‐HER2 (single agent, dual HER2‐targeted therapy, or anti‐HER2 therapy + single‐agent CT/single‐agent HT)	54 (28–85)	198	184	117
Destiny‐Breast02	F. Andrè, The Lancet 2023	III	Open‐lable	HER2+ mBC previously received T‐DM1	T‐DXd (5.4 mg/kg, IV q3w)	54.2 (45.5–63.4)	406	404	406
					Capecitabine (orally 2500 mg/m^2^; 1–14q3w) + trastuzumab (8 mg/kg loading dose followed by 6 mg/kg, IV q3w) Capecitabine (orally 1000 mg/m^2^) + Lapatinib (orally 1250 mg 1‐21q3w)	54.7 (48–63)	202	195	202
Destiny‐Breast04	S. Modi, N Eng J Med 2022 H. Rugo, ESMO Open 2023	III	Open‐label	HER2‐low mBC who had received one or two previous lines of chemotherapy	T‐DXd (5.4 mg/kg, IV q3w)	57.5 (31.5–80.2)	373	371	331
					Capecitabine (orally 2000/2500 mg/m^2^; 1–14q3w) Eribulin (1.23 mg/m2, IV 1,8q3w) Gemcitabine (800–1200 mg/m^2^, IV 1,8,15q4w) Paclitaxel (175 mg/m^2^, IV 1q3w or 80 mg/m^2^, IV q1w) Nab‐paclitaxel (260 mg/m^2^, IV 1q3w or 100 mg/m^2^, IV 1,8,15q4w)	55.9 (28.4–80.5)	184	172	163
Destiny‐Breast06	G. Curigliano, J Clin Oncol 2024	III	Open‐label	ER+/HER2‐low and ultralow mBC chemotherapy‐naïve in the metastatic setting	T‐DXd (5.4 mg/kg, IV q3w)	58.0 (28–87)	436	434	‐
					Capecitabine (orally 2000/2500 mg/m^2^; 1–14q3w) Paclitaxel (175 mg/m2, IV 1q3w or 80 mg/m^2^, IV q1w) Nab‐paclitaxel (260 mg/m^2^, IV 1q3w or 100 mg/m2, IV 1,8,15q4w)	57.0 (32–83)	430	417	–
ASCENT	A. Bardia, N Eng J Med 2021 H. Rugo, Npj Breast Cancer 2022 A. Bardia, J Clin Oncol 2024	III	Open‐label	mTNBC relapsed or refractory to two or more previous standard chemotherapy regimens (no upper limit), previously treated with a taxane (for any indication)	SG (10 mg/kg, IV 1,8q3w)	54 (29–82)	267	258	236
					Capecitabine (orally 2000/2500 mg/m^2^; 1–14q3w) Eribulin (1.23 mg/m^2^, IV 1,8q3w) Gemcitabine (800–1200 mg/m^2^, IV 1,8,15q4w) Vinorelbine (25 mg/m^2^, IV 1q1w)	53 (27–81)	262	224	183
TROPiCS‐02	H. Rugo, Future Oncol 2022 H. Rugo, The Lancet 2023	III	Open‐label	ER+/HER2– mBC treated with 2–4 prior systemic che‐ motherapy regimens for metastatic disease that previously received at least one taxane, at least one anticancer hormonal treatment, and at least one CDK4/ 6i	SG (10 mg/kg, IV 1,8q3w)	57 (29–86)	272	268	236
					Capecitabine (orally 2000/2500 mg/m^2^; 1–14q3w) Eribulin (1.23 mg/m^2^, IV 1,8q3w) Gemcitabine (800–1200 mg/m^2^, IV 1,8,15q4w) Vinorelbine (25 mg/m^2^, IV 1q1w)	55 (27–78)	271	249	210
EVER‐132‐002	B. Xu, Ann Oncol 2023	III	Open‐label	ER+/HER2‐ mBC who had previously received between two and four lines of systemic therapy, treated with at least one endocrine therapy in any setting	SG (10 mg/kg, IV 1,8q3w)	53 (32–72)	166	165	–
					Capecitabine (orally 2000/2500 mg/m^2^; 1–14q3w) Eribulin (1.23 mg/m^2^, IV 1,8q3w) Gemcitabine (800–1200 mg/m^2^, IV 1,8,15q4w) Vinorelbine (25 mg/m^2^, IV 1q1w)	51 (28–79)	165	164	–

Abbreviations: BC breast cancer, CT, chemotherapy; ER, oestrogen receptor; HER2+, HER2‐positive; HT, endocrine therapy; IV, intravenous; TN, triple negative. *Two patients received mistakenly a dose of T‐DM1 thus were included in T‐DM1 group for safety analysis.

### 3.1 Safety outcomes

All nine trials reported data on the frequency of AEs.^2^, ^5^, ^7^, ^17^, ^18^, ^20^, ^21^, ^23^, ^25^

The RRs for each considered AE are summarized for T‐DM1 in Figure 2A, for T‐DXd in Figure 2B and for SG in Figure 2C. The summary of the main AEs evaluated for the analysis and pooled RR for each ADC are described in Table 2, while the corresponding forest plots are available in Figures S1–S12.

**FIGURE 2:**
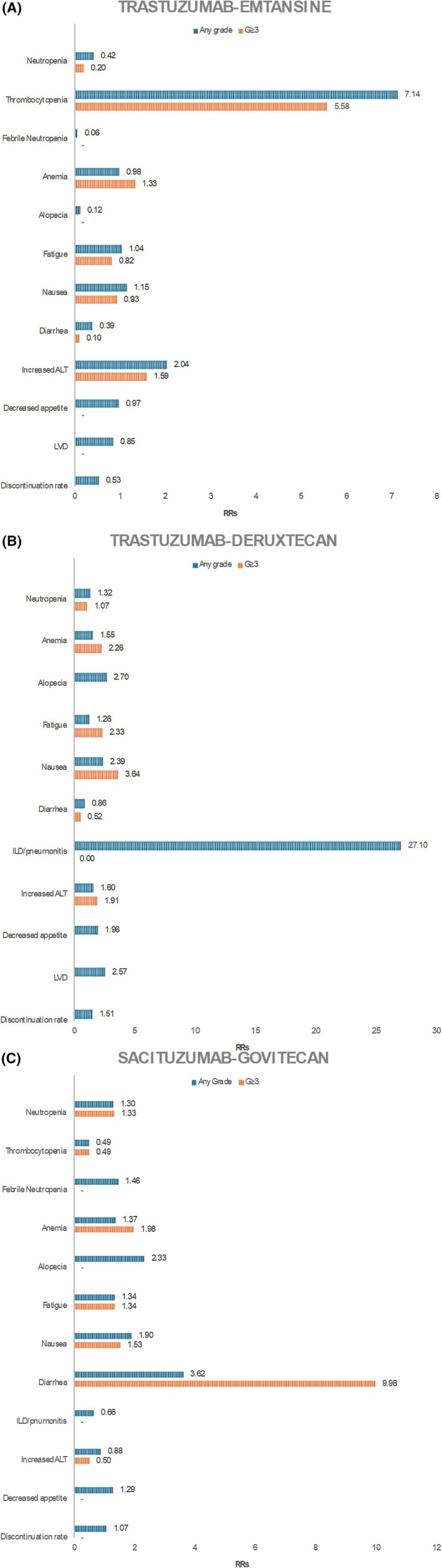
Relative risks of developing AEs of interest per each ADC. (A) Trastuzumab‐emtansine. (B) Trastuzumab‐deruxtecan. (C) Sacituzumab‐govitecan.

**TABLE 2:** Summary of the main AEs evaluated for the analysis and pooled RR for each ADCs.

AEs	ADC	Any grade		≥G3	
		Pooled RR, 95% CI	*I* ^2^	Pooled RR, 95% CI	*I* ^2^
Neutropenia	T‐DM1	.42, .21–.87	86%	.20, .08–.55	79%
	T‐DXd	1.32, .65–2.70	95%	1.07, .34–3.40	96%
	SG	1.30, 1.14–1.49	43%	1.33, 1.12–1.59	45%
Thrombocytopenia	T‐DM1	7.14, 4.13–12.36	40%	5.58, .77–40.26	86%
	SG	.49, .36–.67	0%	.49, .16–1.45	37%
Febrile neutropenia	T‐DM1	–	–	.06, .01–.32	0%
	SG	–	–	1.46, .85–2.51	0%
Anemia	T‐DM1	.98, .52–1.87	79%	1.33, .77–2.30	0%
	T‐DXd	1.55, 1.27–1.90	30%	2.26, 1.47–3.49	0%
	SG	1.37, 1.17–1.60	0%	1.98, 1.27–3.09	0%
Alopecia	T‐DM1	.12, .04–.40	67%	–	–
	T‐DXd	2.70, 1.15–6.30	95%	–	–
	SG	2.33, 1.57–3.47	78%	–	–
Fatigue	T‐DM1	1.04, .76–1.42	78%	.82, .43–1.57	8%
	T‐DXd	1.26, 1.10–1.46	35%	2.33, 1.33–4.09	0%
	SG	1.34, 1.15–1.56	5%	1.34, .42–4.33	76%
Nausea	T‐DM1	1.15, .79–1.69	84%	.93, .17–4.93	53%
	T‐DXd	2.39, 1.90–3.00	74%	3.64, 1.52–8.69	4%
	SG	1.90, 1.65–2.19	0%	1.53, .24–9.70	65%
Diarrhea	T‐DM1	.39, .26–.59	75%	.10, .06–.18	0%
	T‐DXd	.86, .47–1.55	93%	.52, .30–.90	0%
	SG	3.62, 2.97–4.42	0%	9.98, 4.20–23.76	0%
ILD/Pneumonitis	T‐DXd	27.10, 8.67–84.75	0%	–	–
	SG	.66, .05–8.83	28%	–	–
Increased ALT	T‐DM1	2.04, 1.43–2.91	17%	1.59, 0.50–5.11	53%
	T‐DXd	1.60, .87–2.96	88%	1.91, .16–22.20	81%
	SG	.88, .53–1.47	64%	.50, .11–2.37	27%
Decreased appetite	T‐DM1	.97, .75–1.25	24%	–	–
	T‐DXd	1.96, 1.56–2.46	22%	–	–
	SG	1.29, 1.04–1.60	0%	–	–
LVD	T‐DM1	.85, .45–1.60	0%	–	–
	T‐DXd	2.57, 1.68–3.95	0%	–	–

The most common AEs of any grade linked to TDM‐1 included thrombocytopenia (RR 7.14, 95% CI 4.13–12.36) and increased ALT (RR 2.04, 95% CI 1.43–2.91), with also a trend for a higher risk of ≥G3 AEs (RR 5.58,95% CI .77–40.26) and RR 1.59, 95% CI .50–5.11, respectively). For T‐DXd, nausea (RR 2.39, 95% CI 1.90–3.00) and anemia (RR 1.55, 95% CI 1.27–1.90) were the most common AEs of any grade, and also of ≥G3 (RR 3.64, 95% CI 1.52–8.69 and RR 2.26, 95% CI 1.47–3.49, respectively). SG was associated with higher risk of neutropenia any grade (RR 1.30, 95% CI 1.14–1.49), diarrhea (RR 3.62, 95% CI 2.97–4.42) and nausea (RR 1.90, 95% CI 1.65–2.19) as compared to standard therapy. The risk of ≥G3 neutropenia and diarrhea was also higher than with standard treatment (RR 1.33, 95% CI 1.12–1.59 and RR 9.98, 95% CI 4.20–23.76, respectively). Alopecia was also a significant AE to be reported with SG, revealing a twofold increase in the risk compared to standard arm (RR 2.33, 95% CI 1.57–3.47). Severe adverse events such as interstitial lung disease was peculiar to T‐DXd (RR 27.10, 95% CI 8.67‐84.75) ;LVD was more frequent with T‐DXd as compared to T‐DM1 (RR 2.57, 95% CI 1.68–3.95 and RR .85, 95% CI .45–1.60, respectively).

### 3.2 Discontinuation rate due to AEs

All nine trials reported data of discontinuation rate due to AEs.^2^, ^5^, ^7^, ^17^, ^18^, ^20^, ^21^, ^23^, ^25^ No significantly higher rate of discontinuation for any of the ADC included in this analysis was observed, with a trend in favour of T‐DM1 over standard treatment (RR .53, 95% CI .21–1.33) but with high heterogeneity (*I* ^2^ = 90%) (Figure S13). Overall, the ADCs did not seem to be associated with a higher risk of discontinuation due to AEs as compared to standard treatments (Table 3).

**TABLE 3:** Discontinuation rate due to AEs.

	ADC	Trials evaluating the AEs	Pooled RR, 95% CI	*I* ^2^	Overall Pooled RR, 95% CI	*I* ^2^
Discontinuation rate due to AEs	T‐DM1	V. Dieras, 2017 S. Hurvitz, 2013 I. Krop, 2017	.53, .21–1.33	90%	.96, .62–1.49	83%
	T‐DXd	F. Andrè, 2023 H. Rugo 2023 G. Curigliano, 2024	1.51, .87–2.61	79%		
	SG	H. Rugo, 2023 A. Bardia, 2024 B. Xu, 2023	1.07, .66–1.74	0%		

### 3.3 HR‐QoL outcomes

**FIGURE 3:**
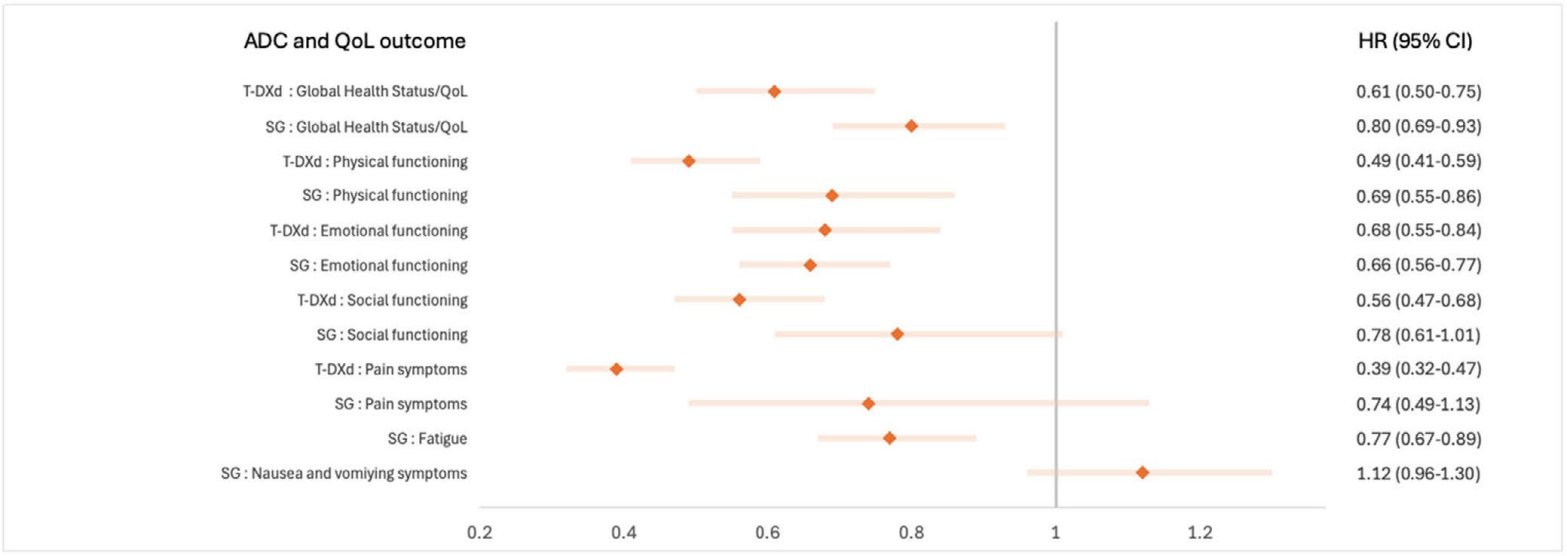
Hazard ratios for each HR‐QoL outcome considered in the analysis per each ADC.

A total of four RCTs were evaluable for the EORTC QLQ‐C30 GHS/QoL TTD analysis.^26^, ^27^, ^28^, ^29^ Treatment with T‐DXd and SG was shown to delay clinical deterioration of GHS over standard therapy, with an HR of .61 (95% CI .50–.75) and .80 (95% CI .69–.93), respectively (Figure 3 and Figure S14.1). T‐DXd and SG also showed to delay clinical deterioration in physical, social and emotional function assessed by EORTC QLQ‐C30 (Figure 3 and Figure S14.2–14.4). TTD of pain was delayed with both T‐DXd and SG (HR .39, 95% CI .32–.47 and HR .74, 95% CI .49–1.13, respectively) (Figure 3 and Figure S14.5). Similar pooled HRs were calculated in the overall analysis.

Only treatment with SG was evaluated for the fatigue as well as the nausea and vomiting symptom scale^26^, ^27^: a significantly delayed fatigue as compared to standard therapy was observed (HR .77, 95%CI .67–.89) (Figure 3 and Figure S14.6). There was a tendency for worse nausea and vomiting with SG compared to standard treatments (HR 1.12, 95% CI .96–1.30) (Figure 3 and Figure S14.7).

## 4 DISCUSSION

To our knowledge, this is the first meta‐analysis that extensively evaluated the risk of AEs as well as HRQoL in RCTs that directly compared ADCs with standard‐of‐care for the treatment of patients with metastatic breast cancer. In the present systematic review and meta‐analysis, we analysed the safety of ADCs currently approved for the treatment of metastatic breast cancer by comparing the most updated results of nine RCTs. As compared to standard therapy, ADCs were globally associated with a higher risk of all‐grade AEs. Thrombocytopenia and increased transaminase levels were the most common AEs associated with T‐DM1, nausea and anemia with T‐DXd, while neutropenia, diarrhea, nausea, fatigue and alopecia with SG. Nevertheless, none of the ADCs were correlated with a significantly higher risk of treatment discontinuation due to AEs compared to the standard of care, nor with a faster deterioration in QoL in almost all domains considered, except for a tendency of worse nausea and vomiting for SG.

The three ADCs differ for structural features and targets: T‐DM1 combines trastuzumab, a HER2‐targeted monoclonal antibody, with the microtubule inhibitor DM1, offering selective cytotoxicity in HER2‐positive cancers. T‐DXd, also an anti‐HER2 targeted agent, utilizes a topoisomerase I inhibitor payload with a cleavable linker, enabling higher drug‐to‐antibody ratios and bystander effects that allows activity also in HER2‐low tumours. In contrast, SG employs an anti‐Trop‐2 monoclonal antibody linked to SN‐38, a potent topoisomerase I inhibitor. While all three ADCs leverage targeted delivery to enhance therapeutic index, their distinct linkers, payloads and targets dictate their clinical applications and efficacy profiles. Moreover, some toxicities appear to be correlated both with the payload itself (i.e. microtubule inhibition disrupting epithelial turnover) or to the result of systemic or localized drug‐related inflammation. Ocular toxicity, for example, although rare, is typical of ADCs: it can manifest through dry eye, keratopathy and blurred vision, described in particular with T‐DM1 and SG.^30^

A recent meta‐analysis of 169 trials evaluated the incidence of AEs with ADCs by considering their payload and target: the targeted agents that most commonly caused all‐grade AEs were anti‐Trop‐2 and anti‐HER2 agents, while DXd, SN‐38 and DM1 were the payload most frequently associated with AEs.^10^ Moreover, AEs correlated to the use of ADCs were reported as significantly more frequent in patients affected by BC than in other tumours.

Despite almost all patients receiving T‐DM1 experienced a transient drop in platelet count, usually it is not associated with severe haemorrhage, reaching a nadir almost 8 days after infusion with a subsequent increase in the values with recovery at day 15.^31^ The mechanisms underlying this phenomenon are likely correlated with the megakaryocytes uptake, with subsequent generation of intracellular catabolite that inhibits platelet production.^32^ According to the recommendations of EMA, T‐DM1 should not be administered with a platelet count <50.000/mm^3^ (G3) and should be prescribed cautiously in patients with ongoing anticoagulant therapy, providing close monitoring.^33^

Regarding the increase in levels of ALT, despite an increased risk of ≥G3 events, it does not frequently impact treatment administration, as most cases are asymptomatic and transient, with slightly increased ALT values with a peak at day 8 and subsequent recovery.^34^ Conversely, a cumulative effect has also been observed, improving with dose reduction. According to the label, treatment should be withdrawn in cases of grade 3 or higher elevation.^33^

The recent implementation of T‐DXd in clinical practice has significantly transformed the treatment landscape for both HER2‐positive and HER2‐low advanced breast cancer. However, this advancement comes with the challenge of managing distinct severe AEs that need to be carefully addressed. Among the common AEs reported in RCTs, nausea appeared to be the most frequent. Therefore, administering appropriate prophylaxis for acute and delayed nausea is crucial to prevent and manage these events. Current guidelines consider T‐DXd as a high emetogenic agent, thus suggesting a prophylactic combination of three drugs including dexamethasone, a 5‐hydroxytryptamine (5‐HT3) receptor antagonist along with a neurokinin‐1 receptor antagonist.^35^ For patients who experience nausea or vomiting despite the three‐drug regimen, the addition of olanzapine should be taken into consideration.^35^

Regarding toxicities of special interest correlated to T‐DXd, ILD is particularly noteworthy and is explicitly highlighted as a warning on the approved label. Our meta‐analysis revealed a significative increased risk of developing ILD, concordant with previous data (10.4% in DestinyBreast‐02 trial^5^ and 12.1% in DestinyBreast‐04 trial^4^, with a total of two and three fatal events, respectively). Given the frequency and severity of this phenomenon, both clinicians and patients should be educated to early identify and manage this event. Regular monitoring is warranted for patients treated with T‐DXd by using CT scan of the chest at the treatment initiation and at least every 9–12 weeks during drug exposure.^36^ For patients developing ILD, it is recommended to suspend treatment and have them evaluated by a pulmonary specialist, with follow‐up imaging. Prompt steroid therapy should be prescribed. Rechallenging with T‐DXd may be considered in selected cases, such as grade 1 ILD/pneumonitis after resolution,^36^ taking into consideration that the risk of developing a second ILD for these patients is approximately 30%.^37^

Regarding SG, diarrhea was the most common AE reported in RCTs, with almost a three‐fold increase in the risk of any‐grade diarrhea. This AE can be severe, thus proper management is essential.

Our meta‐analysis showed a higher risk of neutropenia any grade and severe with SG as compared to standard therapy. These findings underscore the critical need to address these potentially life‐threatening events. The treatment with SG should not be administered in case of absolute neutrophil count below 1500/mm^3^.

Routine primary prophylaxis with granulocyte‐colony stimulating factors (G‐CSF) is not usually recommended, but its administration could be considered as secondary prophylaxis at the first occurrence of G4 neutropenia with a duration of more than 7 days, febrile neutropenia, or G3–4 neutropenia that has delayed dosing by 2 or 3 weeks for recovery to ≤ G1.^38^

Moreover, we previously highlighted alopecia as an emergent significant adverse event with SG, likely due to its payload, SN‐38.^39^ Patients should be informed about this side effect. Scalp cooling has not yet been studied in this population, so further research is necessary to evaluate its value.

Despite a higher risk of developing most of the considered AEs than standard therapy, we did not observe a significant increase in the discontinuation rate with the use of the evaluated ADCs . However, this result should be considered with caution taking into account the different positioning of the ADCs within the history of the disease in the included trials.

The evaluation of PROs in oncology trials is a crucial method for examining how anticancer treatments affect specific symptoms and HRQoL. This evaluation also helps improving the counselling on the balance between the risks and benefits of a specific treatment.

Our meta‐analysis showed that T‐DXd and SG as compared to standard therapy were associated with a significantly delayed deterioration in different QoL domains, especially regarding physical, emotional functioning, social functioning and pain symptoms. These findings are consistent with a prior meta‐analysis of 12 RCTs involving different tumour types, including metastatic breast cancer, showing that treatment with ADCs significantly delayed the deterioration of patients' clinical condition compared to chemotherapy.^40^ Hence, along with greater clinical activity, ADCs have shown to provide a more favourable impact on daily well‐being across studies.

On the other hand, the impact of SG on the rise of the risk of developing nausea and vomiting highlights the importance of giving proper antiemetic prophylaxis.

Conversely, a recent survey revealed that physicians are not always aware of the emetogenicity of ADCs, especially when not frequently users, with subsequent less frequent prescription of proper prophylaxis.^41^ According to current guidelines, SG is considered a high emetogenic agent with a median onset of nausea of about 1 week after infusion.^22^, ^35^ An adequate prevention of acute‐ and delayed‐onset of nausea is recommended, by providing the use of schemes of a combination of drugs that include 5HT‐3 receptor antagonist, corticosteroids and neurokinin 1 (NK1) receptor antagonist.^35^, ^42^ Taken together, these findings endorse the superiority of SG over standard therapy in terms of QoL benefit among all the domains investigated, except for nausea and vomiting. On the contrary, despite nausea represents a major AE reported with T‐DXd, we lack data on the impact on the QoL correspondent domain as it was not assessed in the evaluation of PROs related to T‐DXd use.

Our study has some limitations. Firstly, it is important to note that the RCTs included in our analysis investigated ADCs at different stages of the disease, with a higher risk of AEs for highly pre‐treated patients. When translating these results into clinical practice, it should be noted that the data were collected from RCTs involving highly selected patients with limited comorbidities and thus cannot be precisely applied to the real‐world setting.

## 5 CONCLUSIONS

The present meta‐analysis quantifies the risk of developing different AEs with the administration of the currently approved ADCs in the treatment of metastatic breast cancer. By aggregating data from multiple studies, this analysis provides a comprehensive understanding of the toxicity profile of these agents, thereby enabling healthcare professionals to better assess and manage these risks in clinical practice. The data on treatment discontinuation and HR‐QoL reinforce the positive risk–benefit profile of these ADCs. As the therapeutic landscape expands with new trials involving novel ADCs targeting different molecules and using various payloads, it becomes essential to investigate in depth ADC toxicities to improve their management enhancing the well‐being and QoL of patients.

## AUTHOR CONTRIBUTIONS

The authors confirm the contribution to the paper as follows: study conception and design, analysis and interpretation of the results: MP, EB and ML; data collection: MP and CM; draft manuscript preparation: MP, EB, CM, TR, IG, LA, SN, MGR, RB, DF, CL, EC, LT, EM, KJ, MS, SO, GS, LML, SS, CS and ML. All authors reviewed the results and approved the final version of the manuscript.

## CONFLICT OF INTEREST STATEMENT

MP reported honoraria from Daiichi Sankyo. EB reported receiving research funding (to her institution) from Gilead, speaker fees from Eli Lilly. CS reports consulting fees, honoraria or meeting/travel support from AstraZeneca, Boehringer Ingelheim, Bristol Myers Squibb, Daiichi Sankyo, Eisai, Genentech,Gilead, Lilly, MediTech, Menarini, MSD Spain, Novartis, Pfizer, Philips Healthcare, Pharmalex, Pierre Fabre, Puma Biotechnology, Roche, Seagen, Synthon and Zymeworks.ML: advisory role for Roche, Lilly, Novartis, Astrazeneca, Pfizer, Seagen, Gilead, MSD, Exact Sciences, Pierre Fabre, Menarini; speaker honoraria from Roche, Lilly, Novartis, Pfizer, Sandoz, Libbs, Daiichi Sankyo, Takeda, Menarini, AstraZeneca; travel Grants from Gilead, Daiichi Sankyo, Roche; research funding (to the Institution) from Gilead all outside the submitted work.

## Supporting information


Figures S1–S14.S

## Data Availability

The authors confirm that the data supporting the findings of this study are available within the article and its supplementary materials.
