# Supplementary material for: Adverse events and impact on quality of life of antibody‐drug conjugates in the treatment of metastatic breast cancer: A systematic review and meta‐analysis
Source: Eur J Clin Invest. 2025 Feb 13;55(6):e70001. doi: 10.1111/eci.70001 (PMC12066883; doi:10.1111/eci.70001)
Supplement: Supplementary file 1 — Figures S1–S14.S [file ECI-55-e70001-s001.docx]

**Figure S1. Forest plot of RR of neutropenia.**

| **S1.1 Neutropenia any grade**  **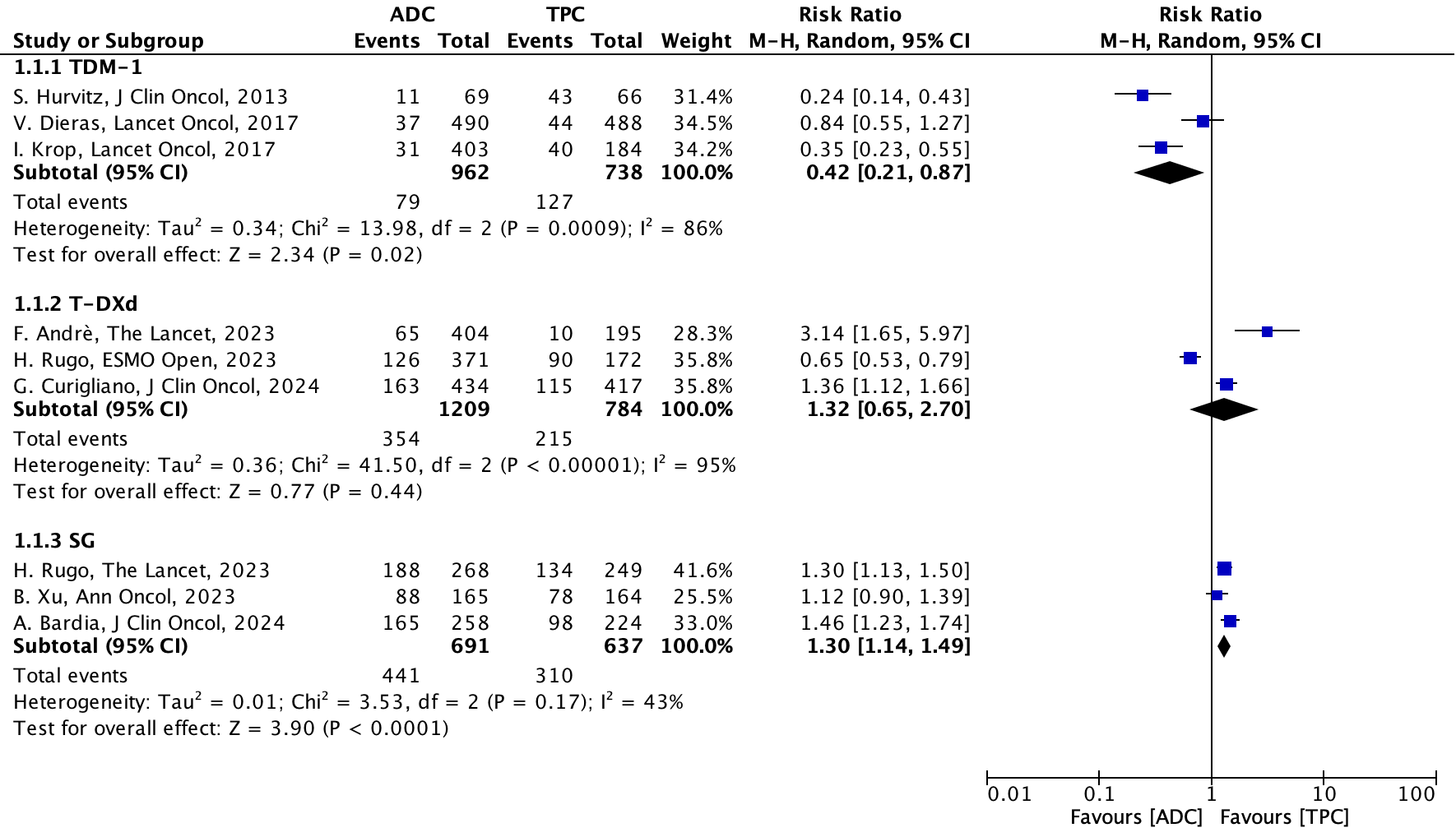** |
| --- |
| **S1.2 Neutropenia ≥G3**  **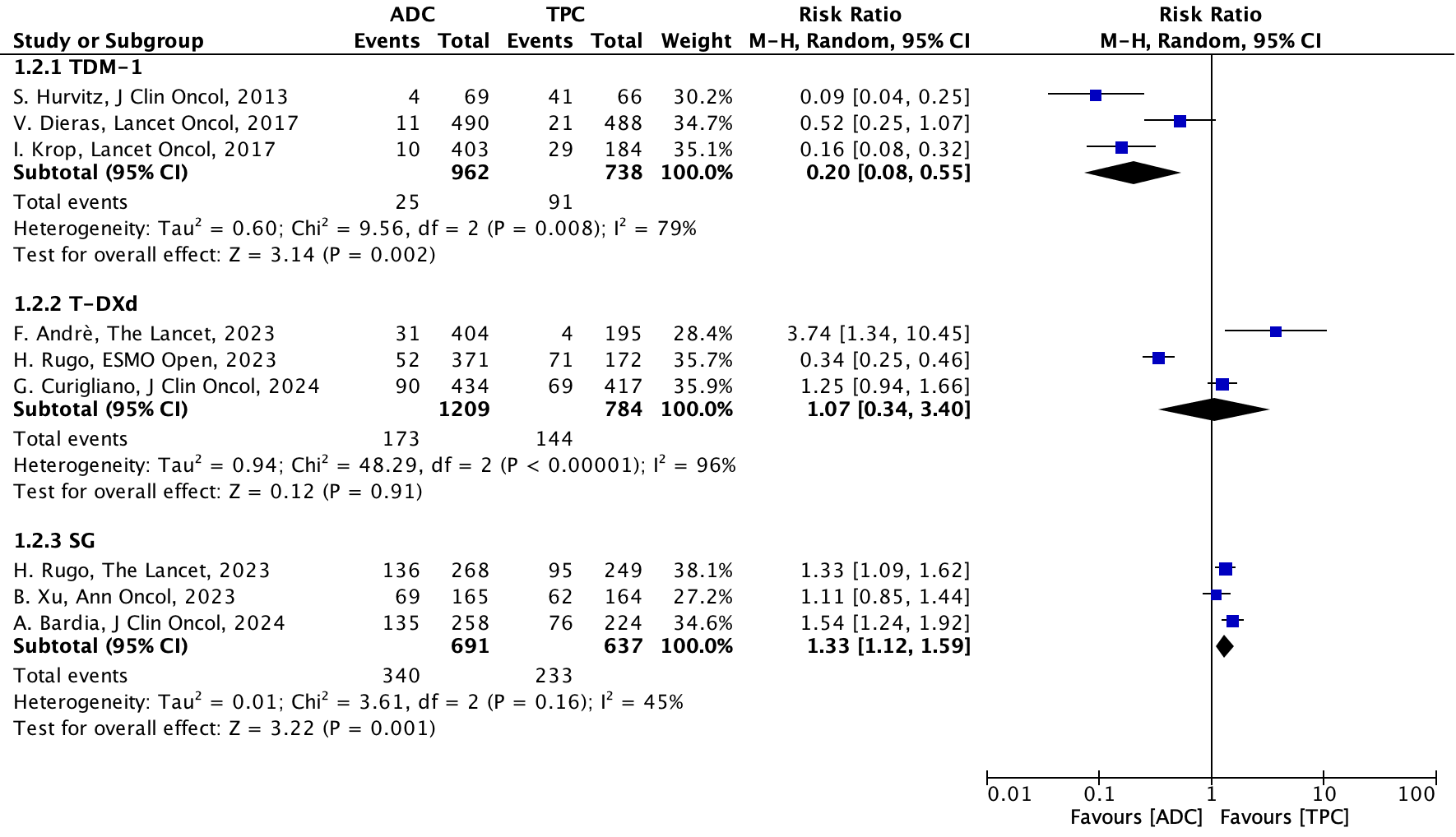** |

**Figure S2. Forest plot of RR of thrombocytopenia.**

| **S2.1 Thrombocytopenia any grade**  **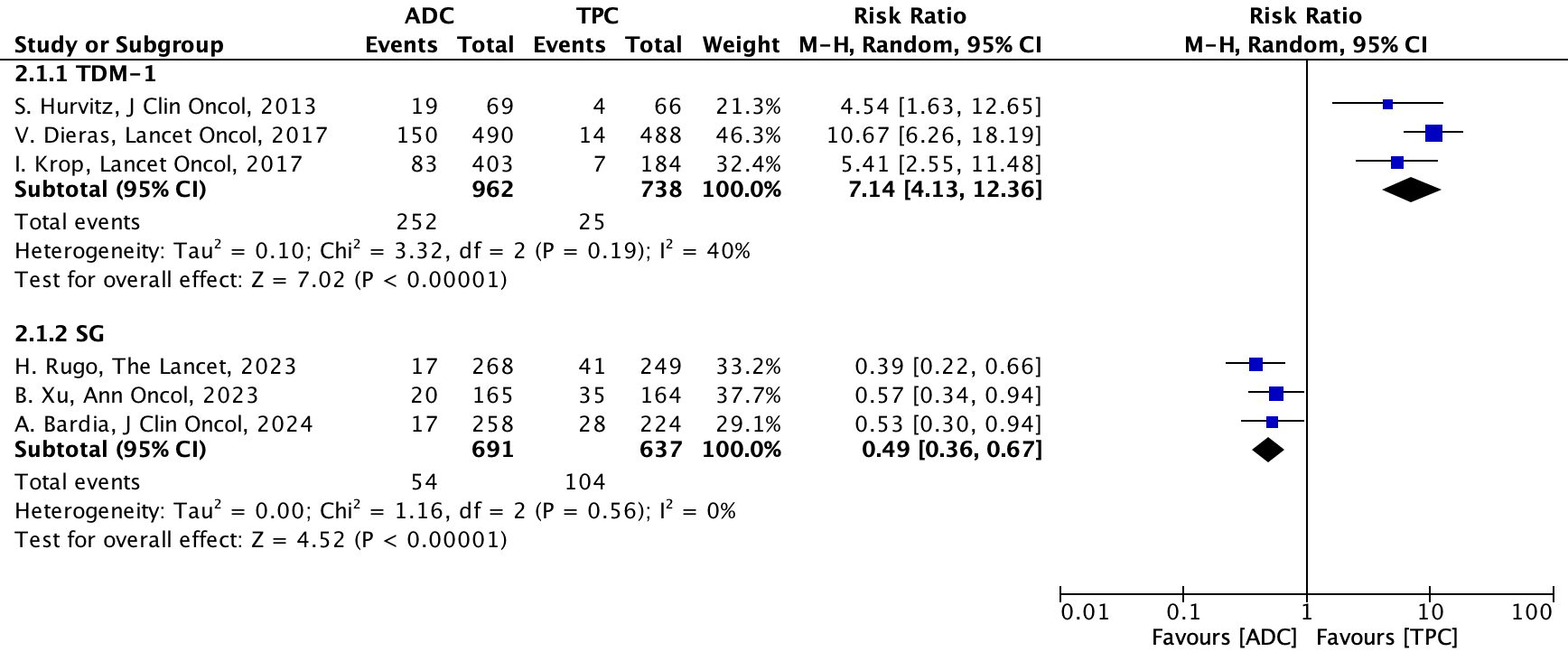** |
| --- |
| **S2.2 Thrombocytopenia ≥G3**  **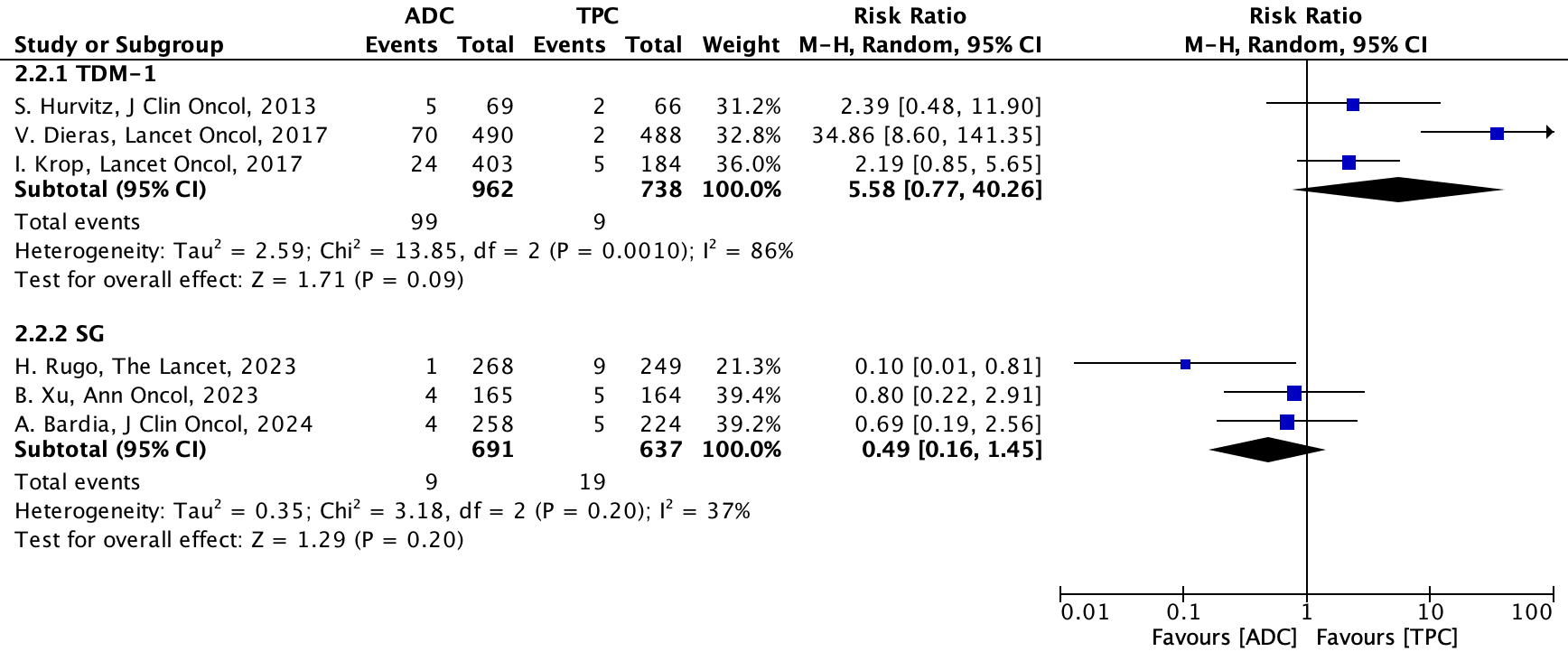** |

**
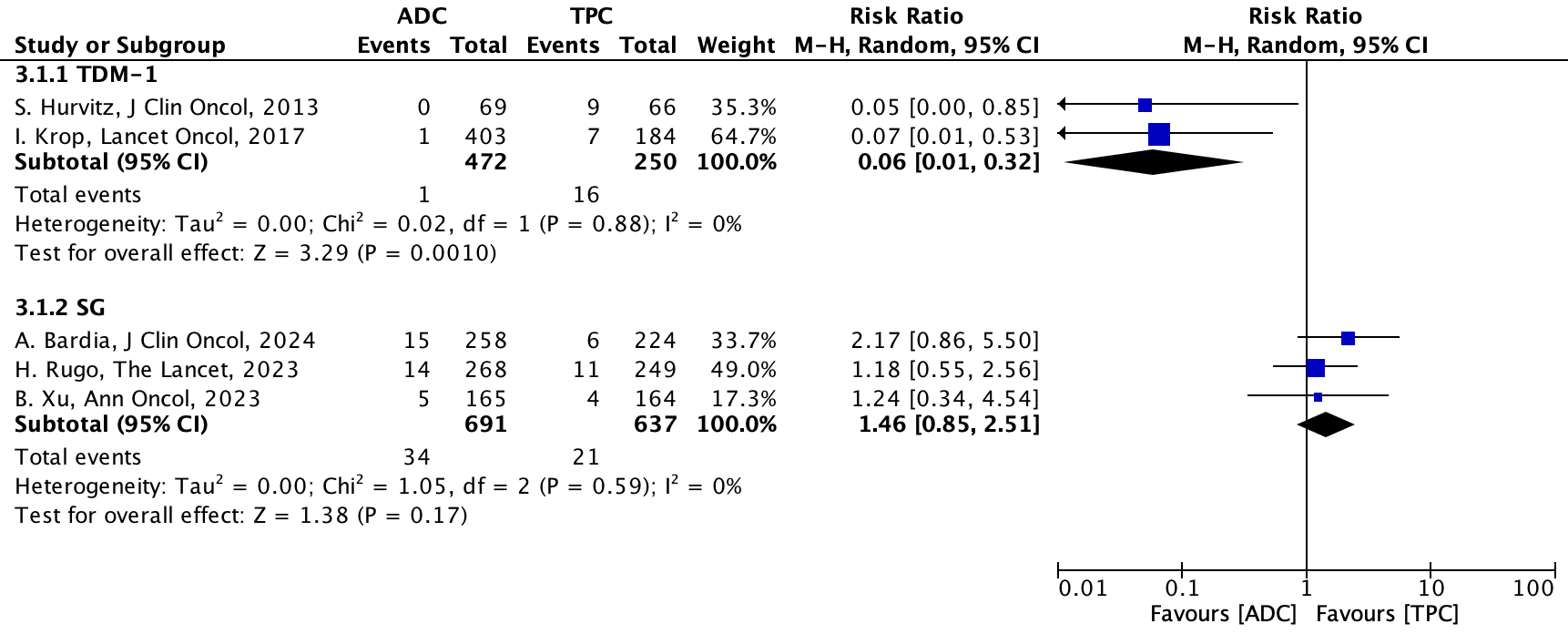
Figure S3. Forest plot of RR of febrile neutropenia.**

**Figure S4. Forest plot of RR of anemia.**

| **S4.1 Anemia any grade**  **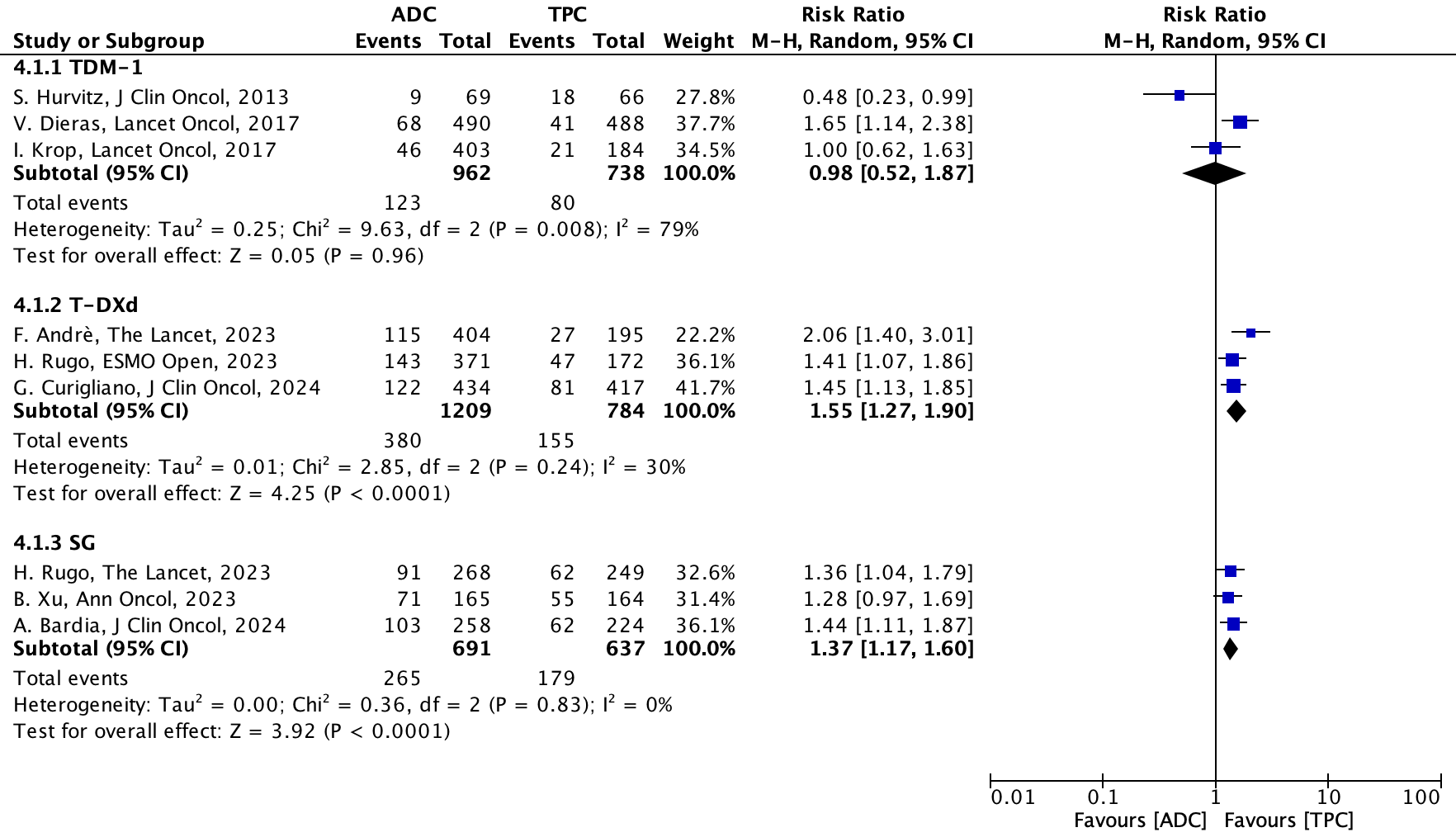** |
| --- |
| **S4.2 Anemia ≥G3**  **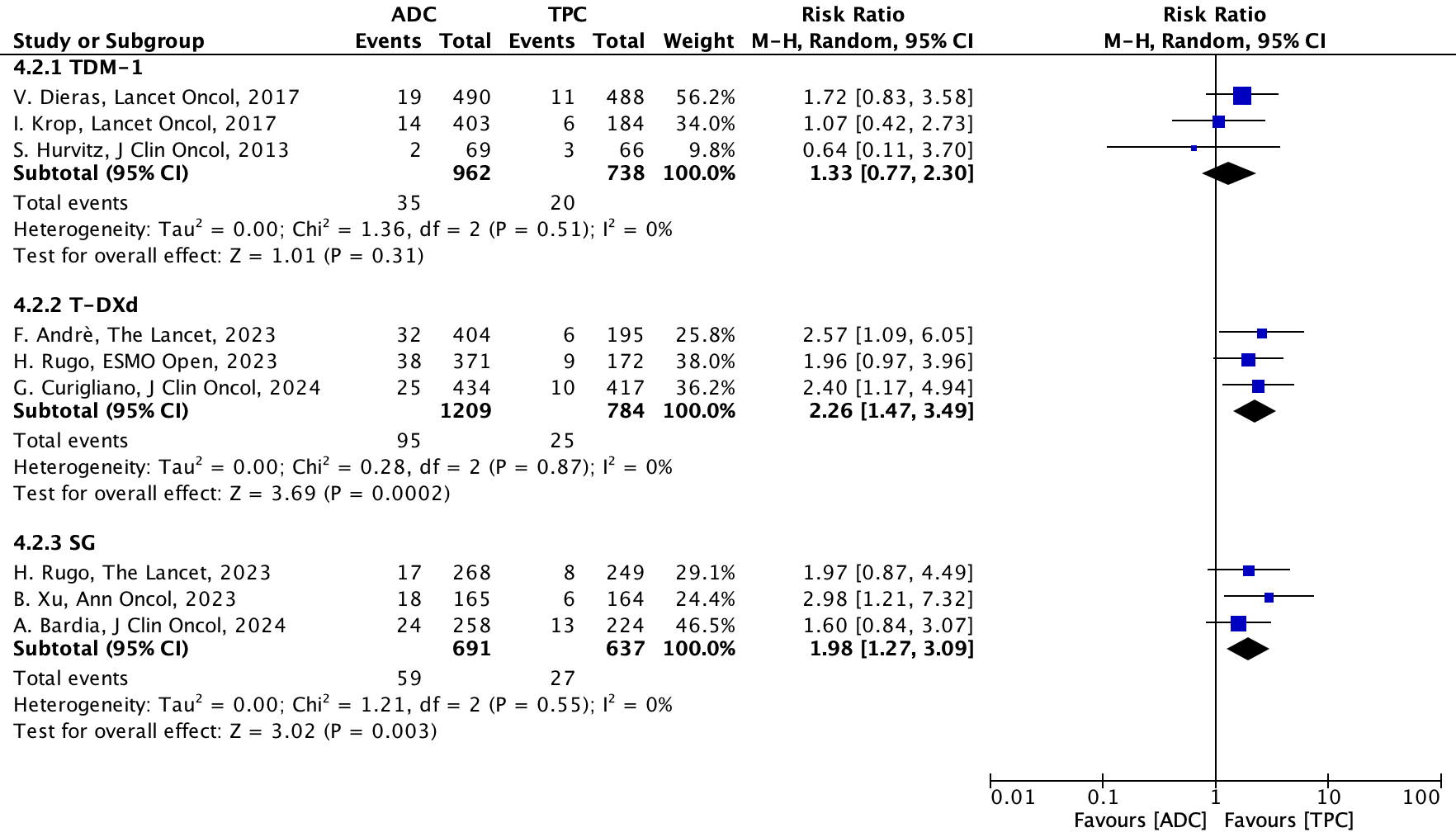** |

**
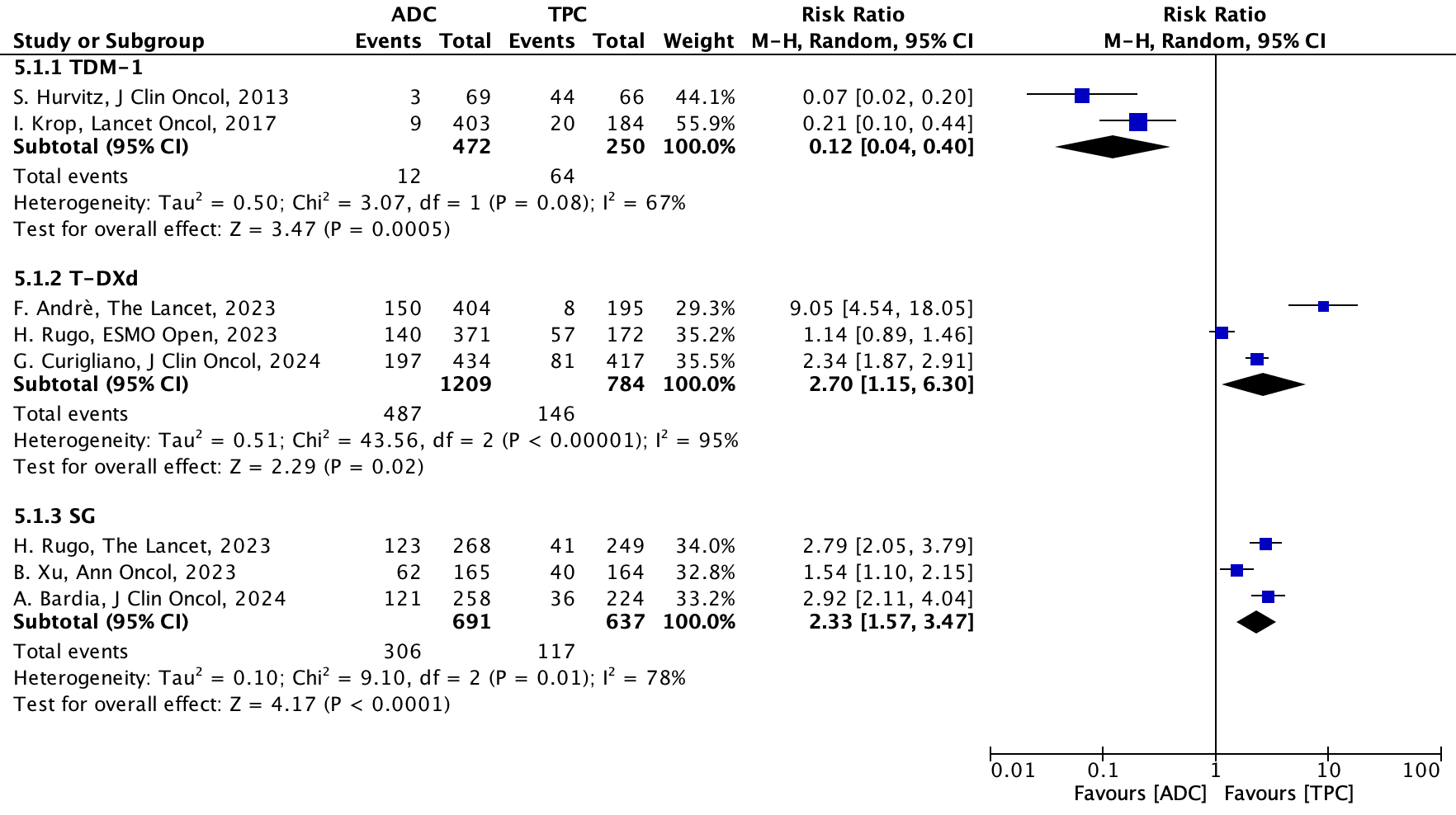
Figure S5. Forest plot of RR of alopecia.**

| **S6.1 Fatigue any grade**  **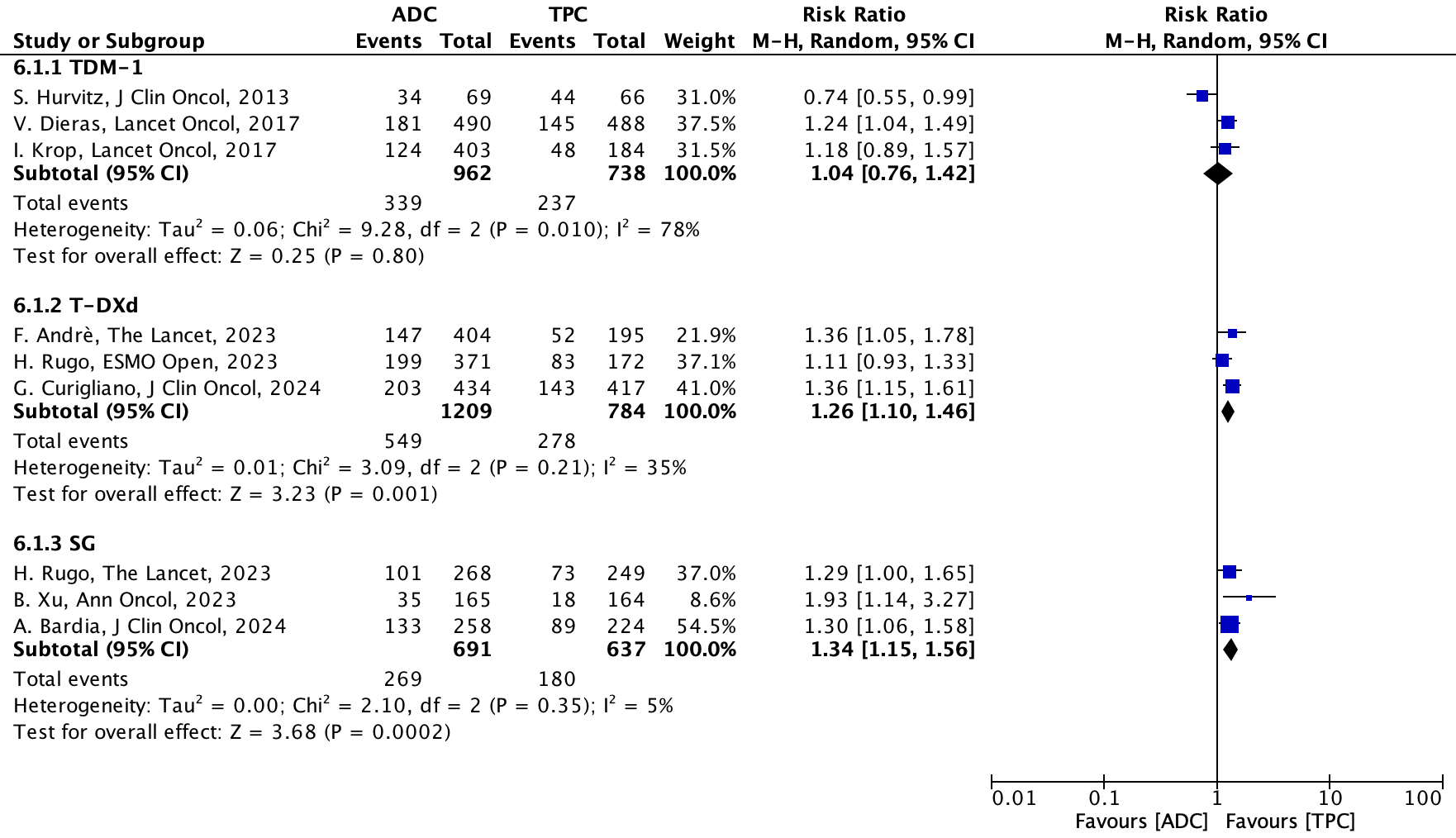** |
| --- |
| **S6.2 Fatigue ≥G3**  **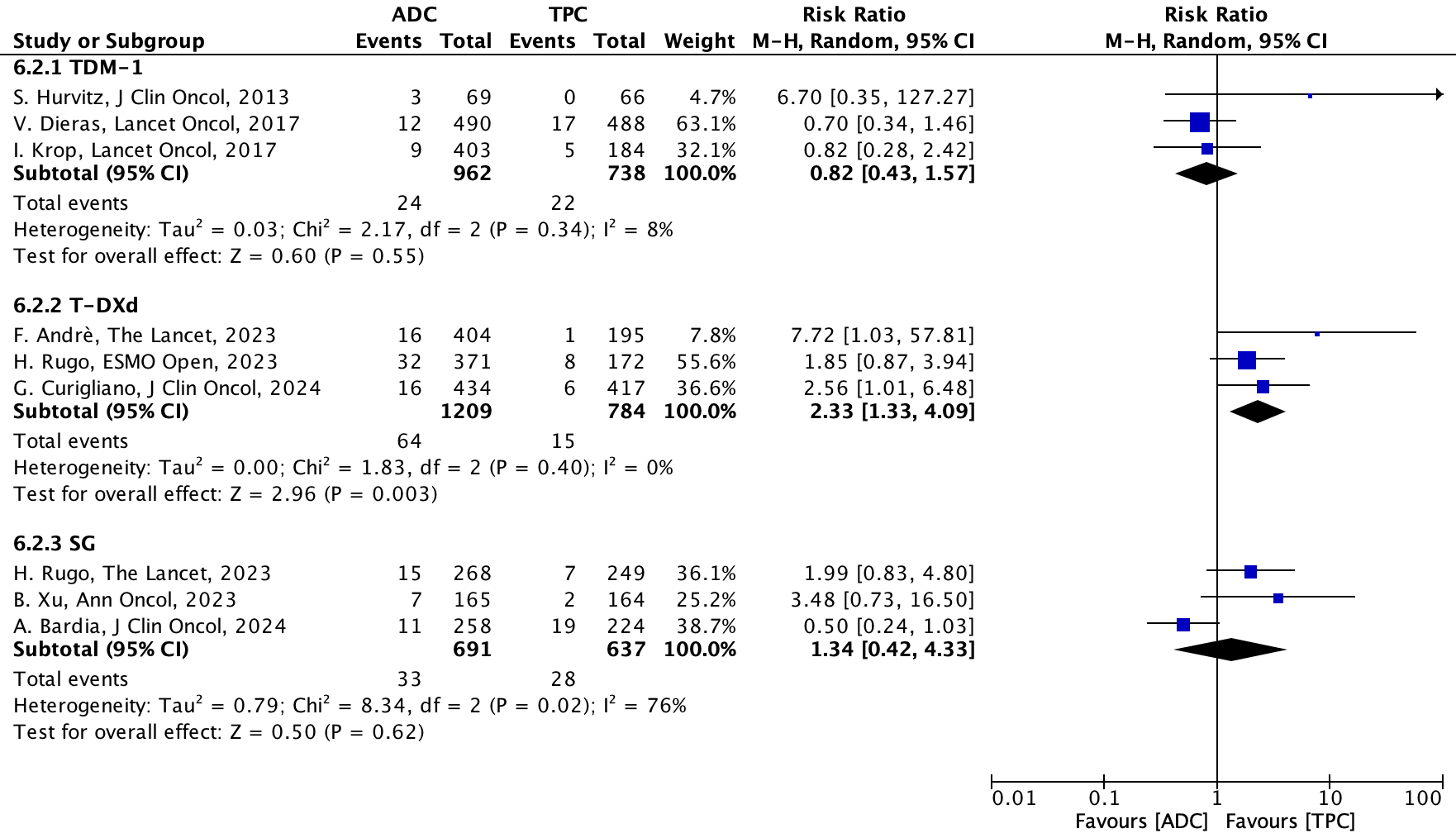** |

**Figure S6. Forest plot of RR of fatigue.**

**Figure S7. Forest plot of RR of nausea.**

| **S7.1 Nausea any grade**  **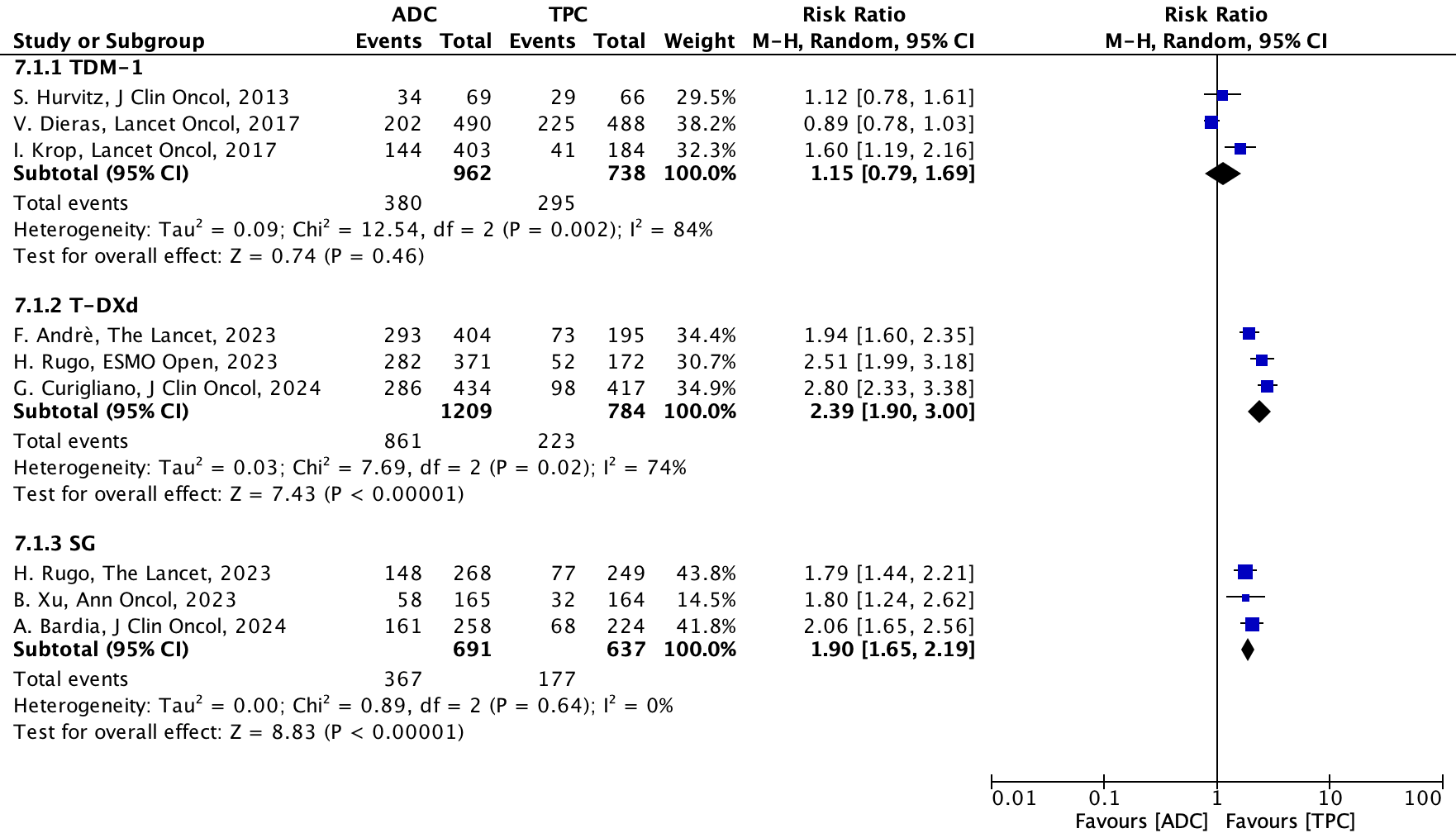** |
| --- |
| **S7.2 Nausea ≥G3**  **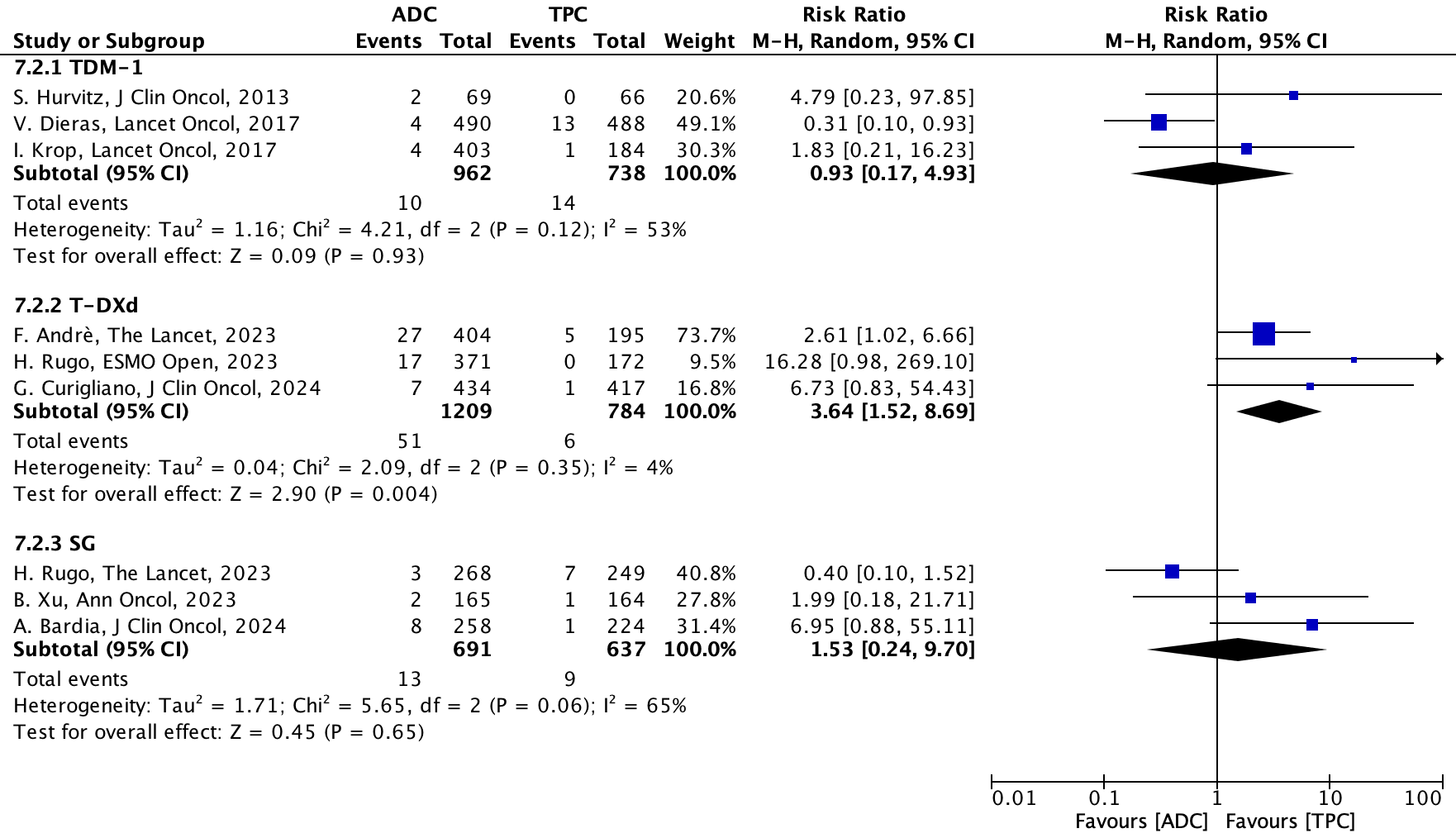** |

**Figure S8. Forest plot of RR of diarrhea.**

| **S8.1 Diarrhea any grade**  **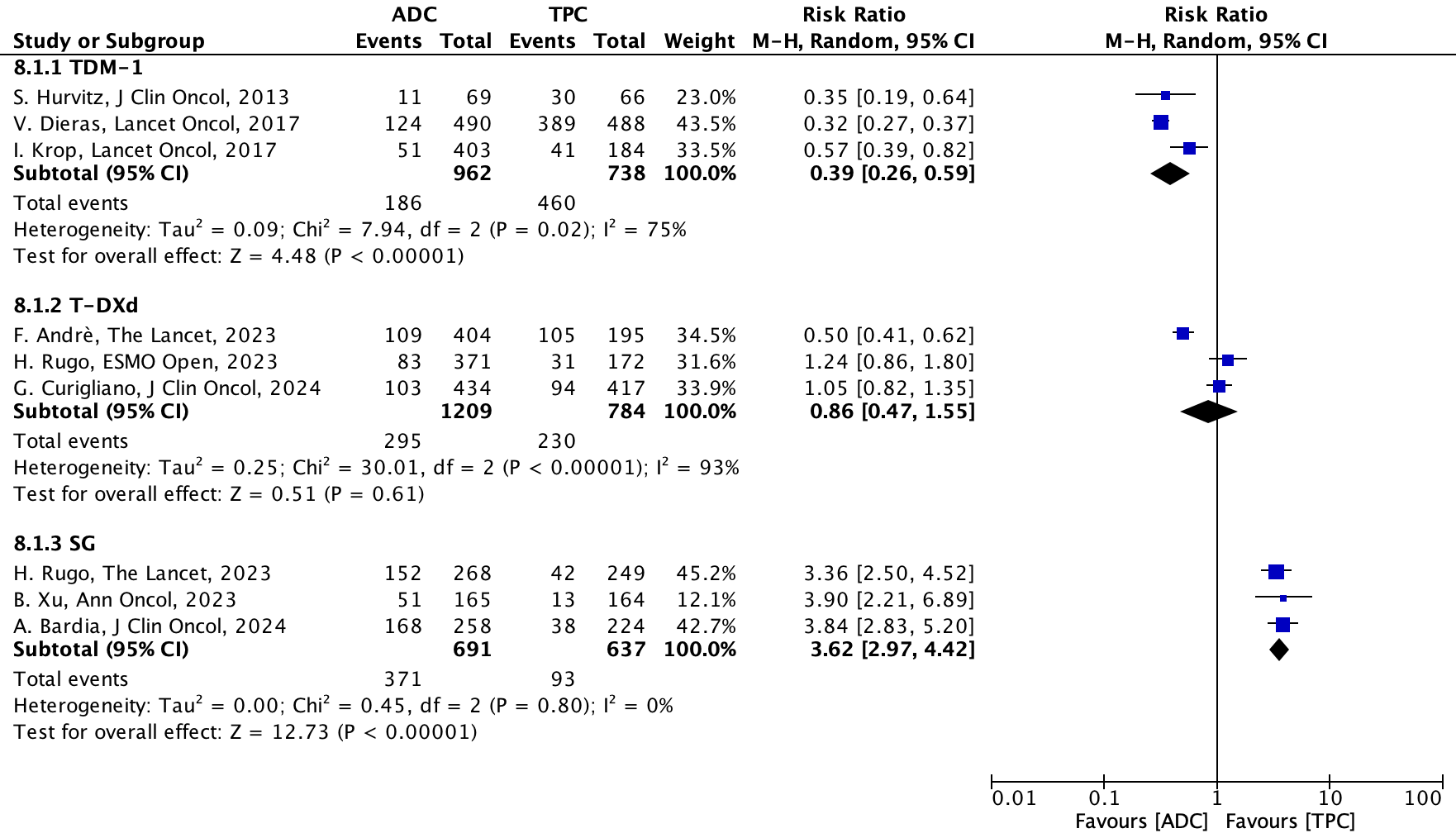** |
| --- |
| **S8.2 Diarrhea ≥G3**  **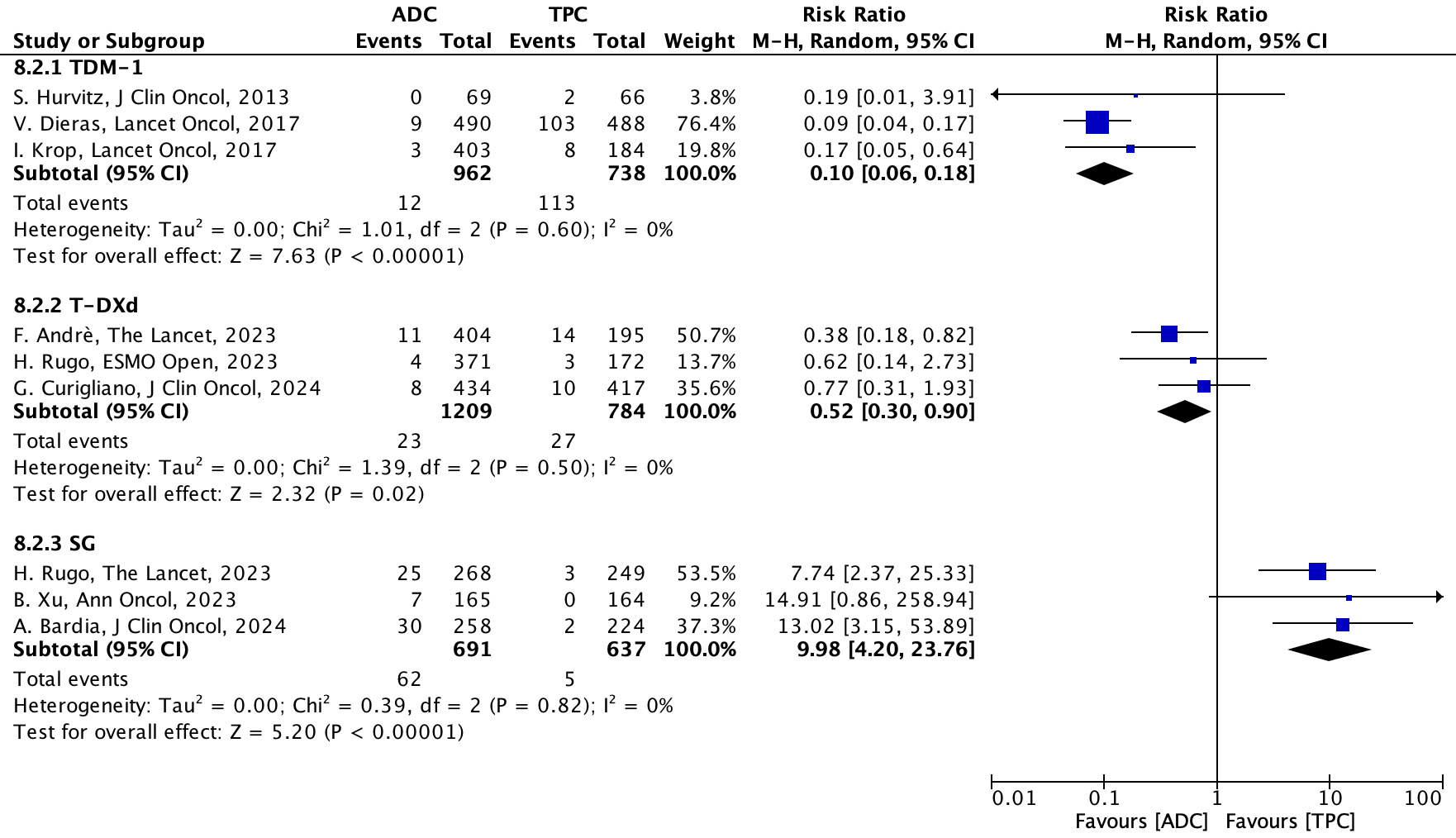** |

**Figure S9. Forest plot of RR of ILD/Pneumonitis.**


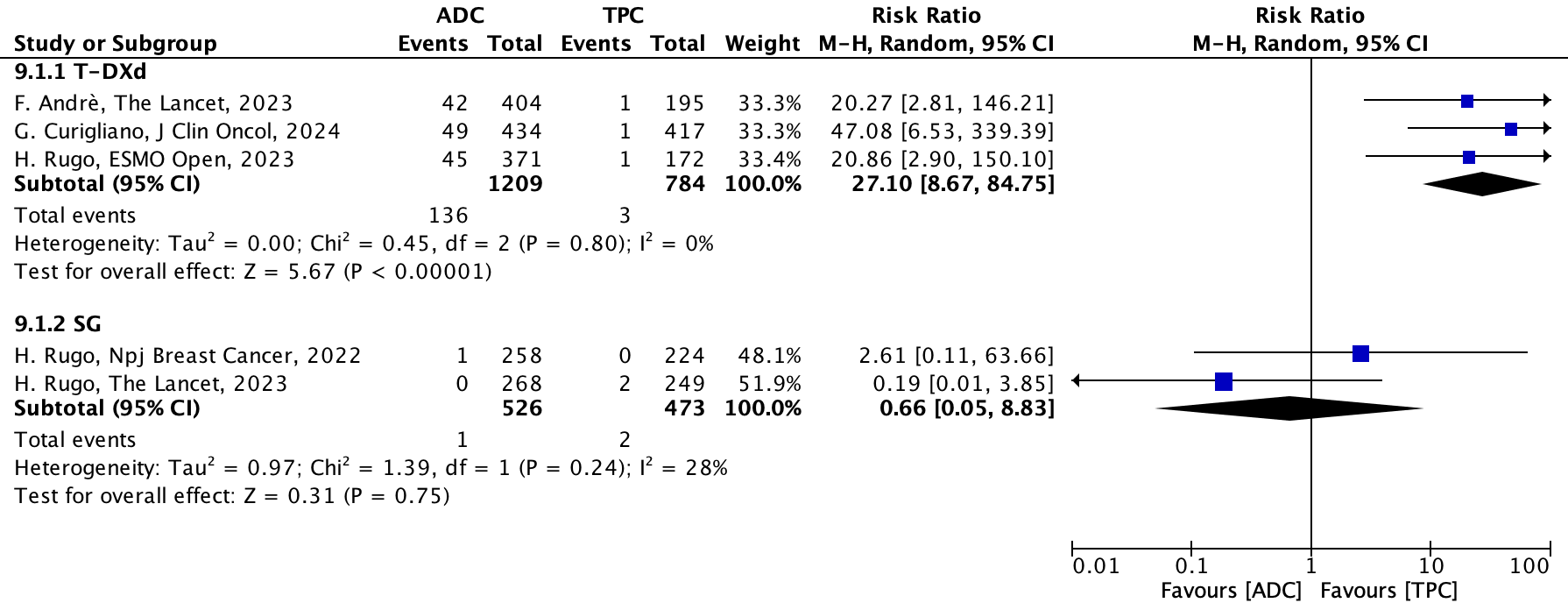


**Figure S10. Forest plot of RR of increased ALT.**

| **S10.1 Increased ALT any grade**  **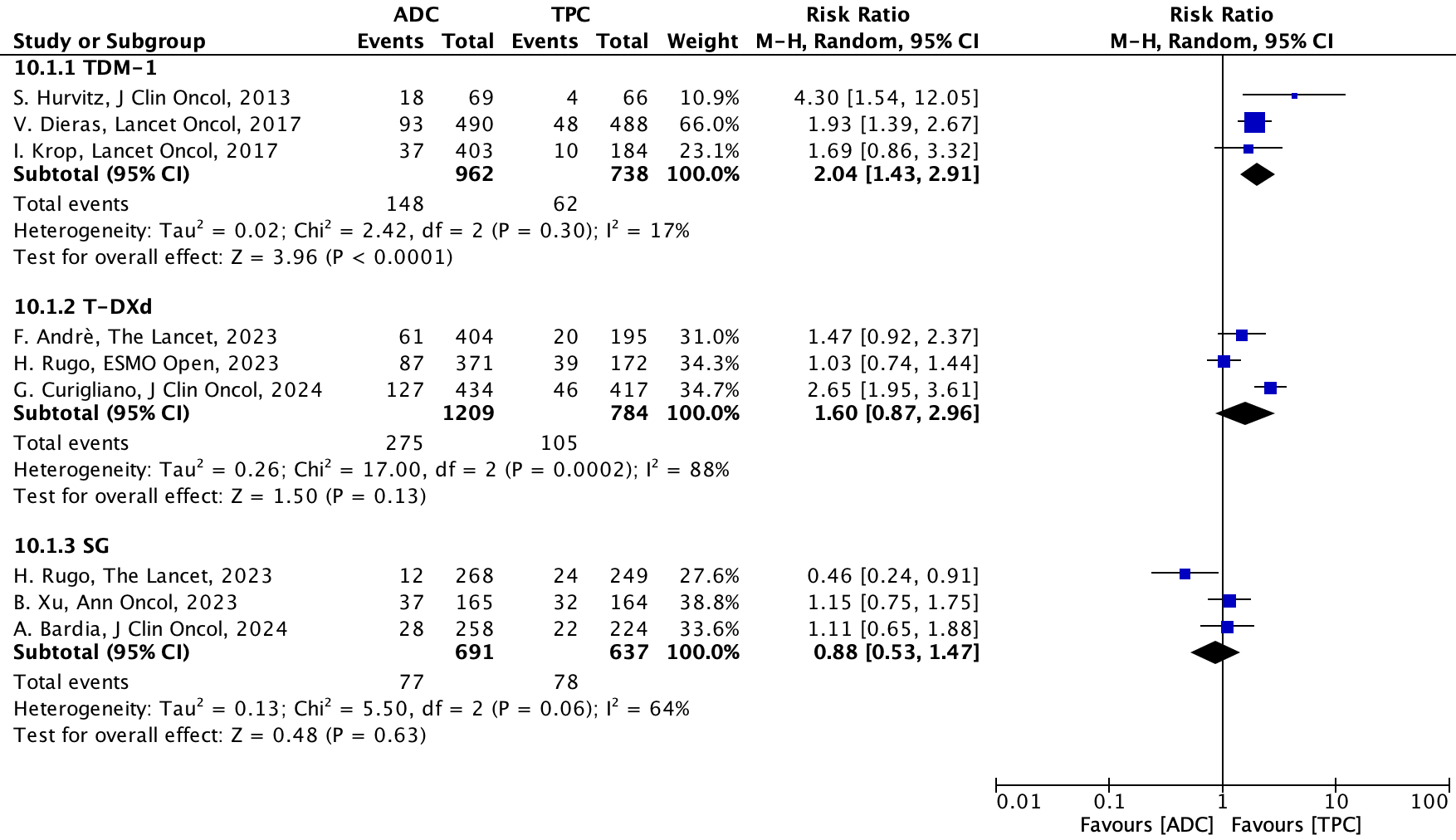** |
| --- |
| **S10.2 Increased ALT ≥G3**  **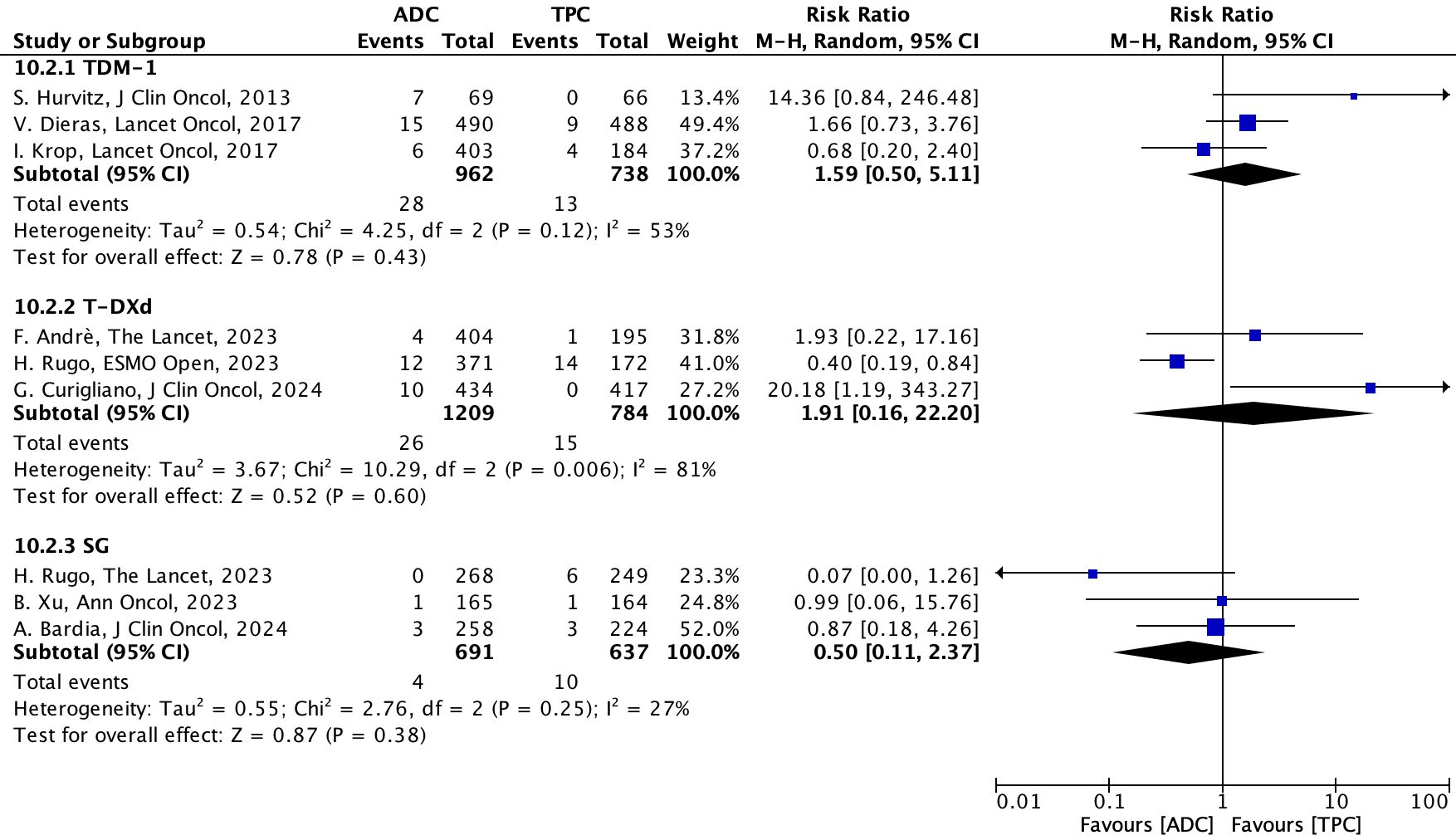** |

**Figure S11. Forest plot of RR of decreased appetite.**

**
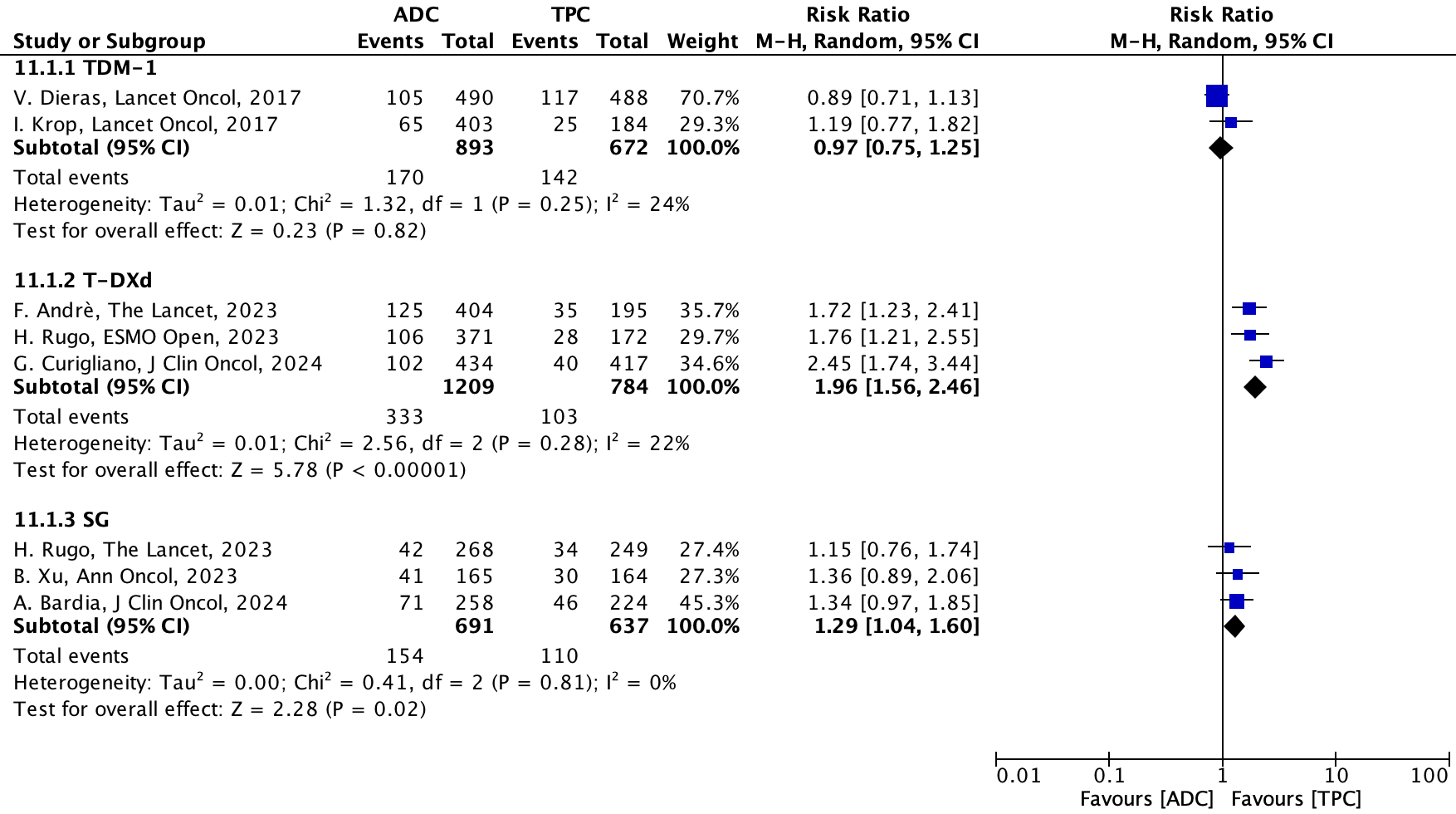
**

**Figure S12. Forest plot of RR of LVD.**

**
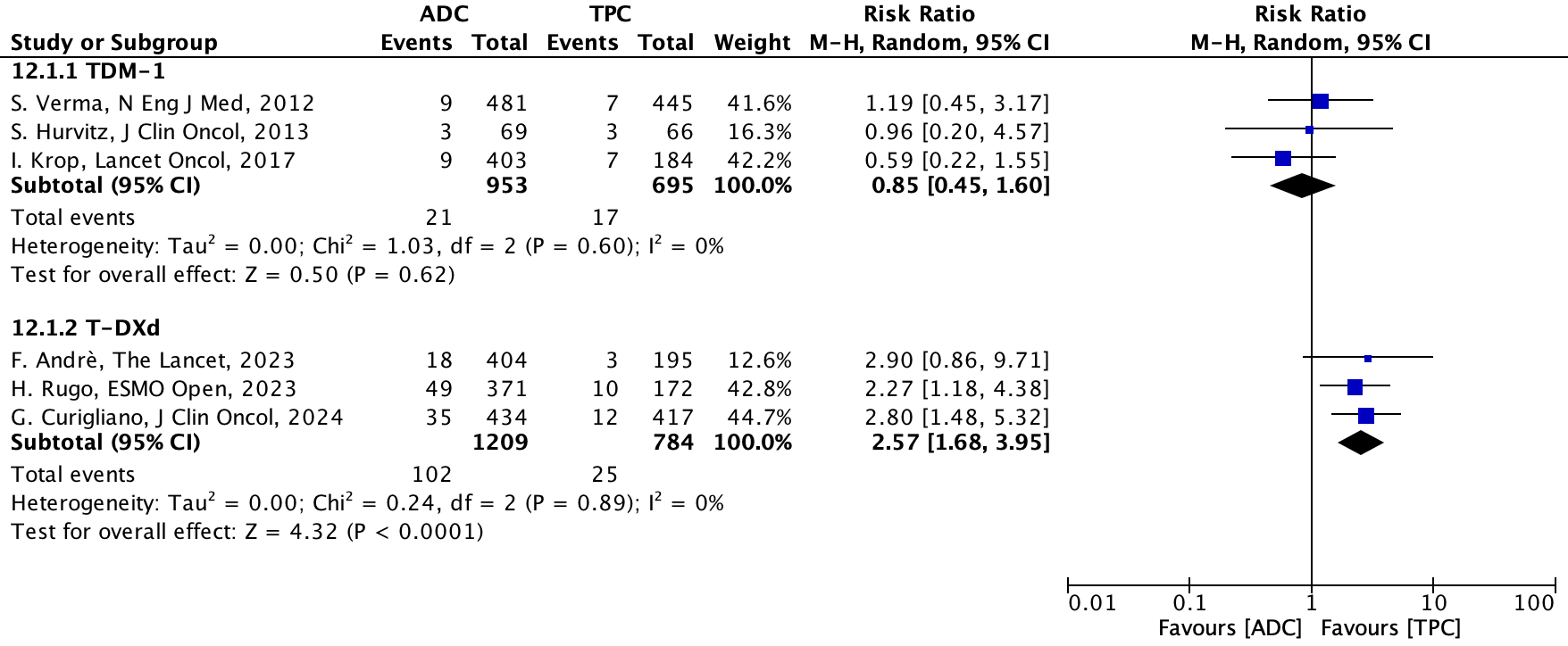
**

**Figure S13. Forest plot of DR.**

**
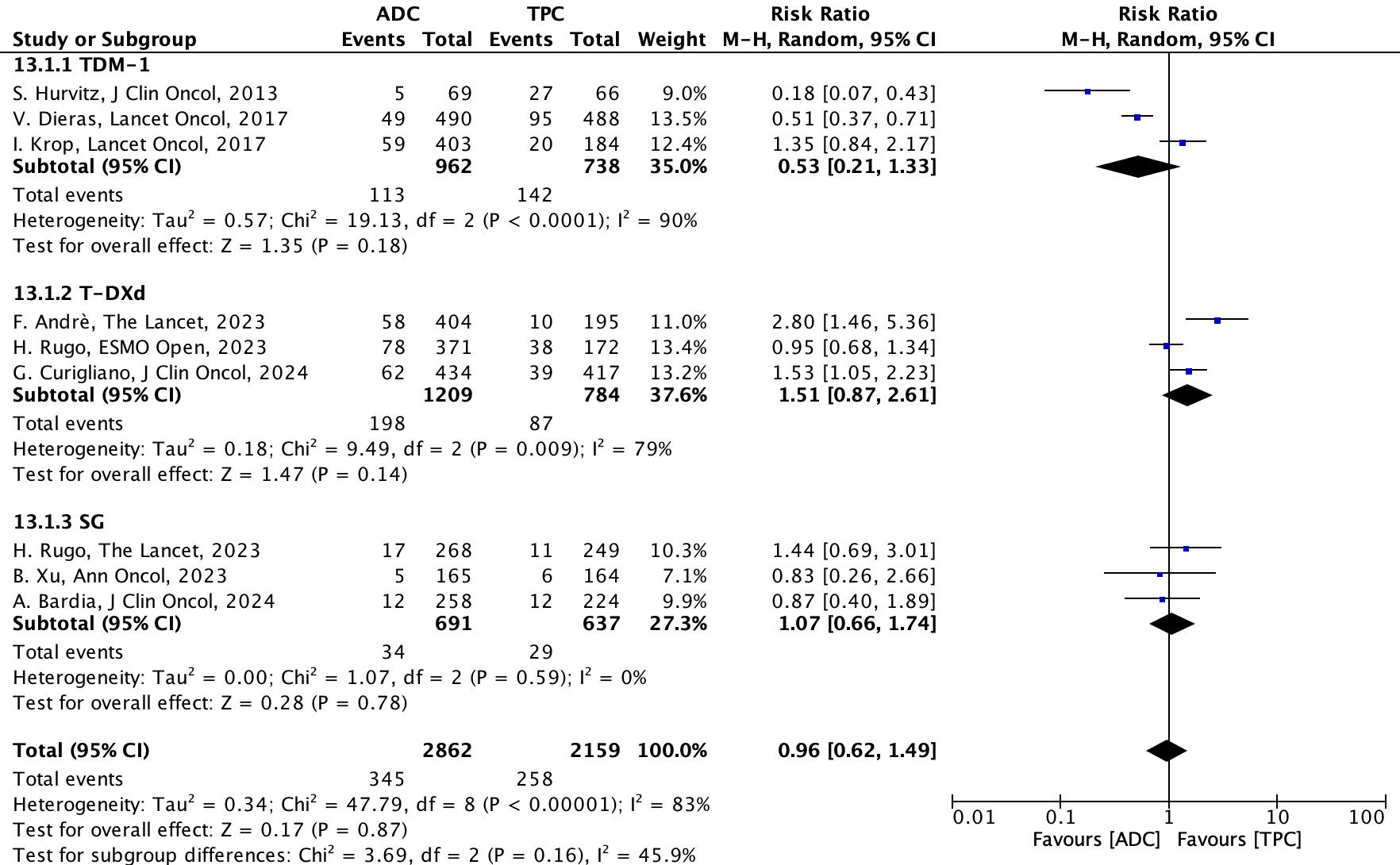
**

**Figure S14.1 Forest plot of HR of TTD according to EORTC QLQ-C30, outcome: Global Health Status/QoL.**

**
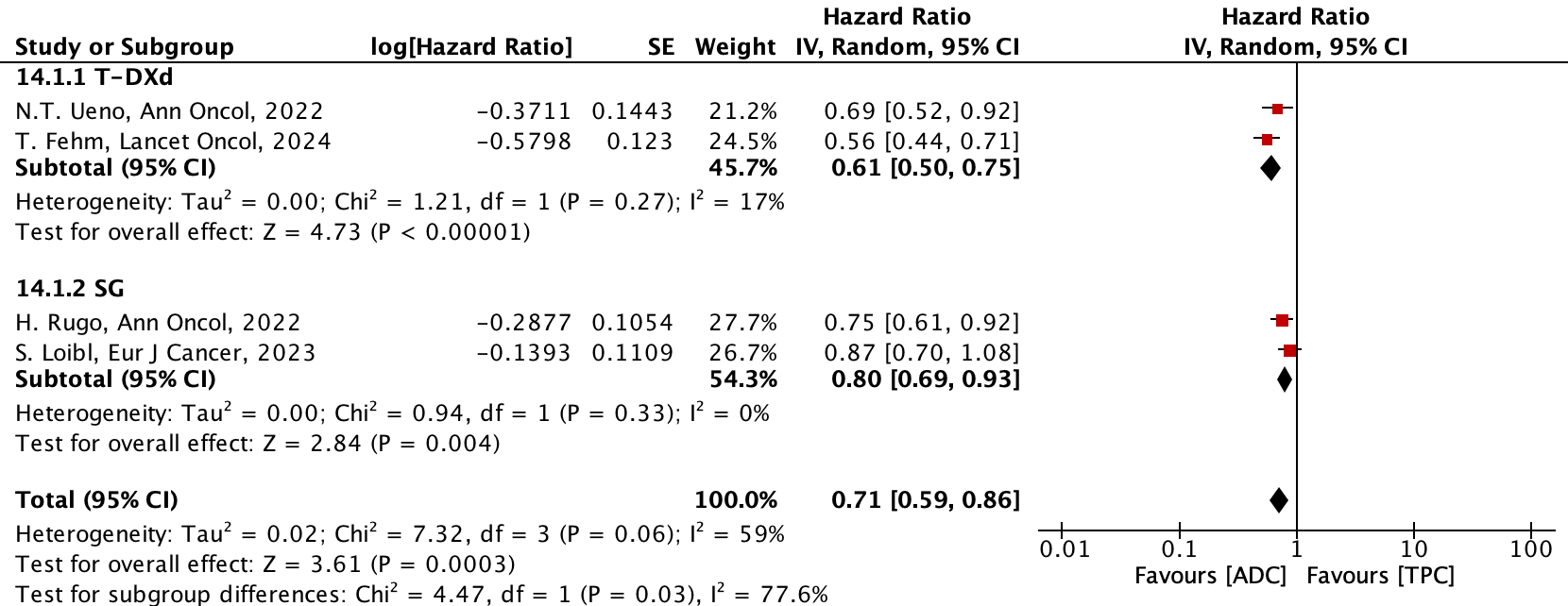
**

**Figure S14.2 Forest plot of HR of TTD according to EORTC QLQ-C30, outcome:** **physical functioning.**


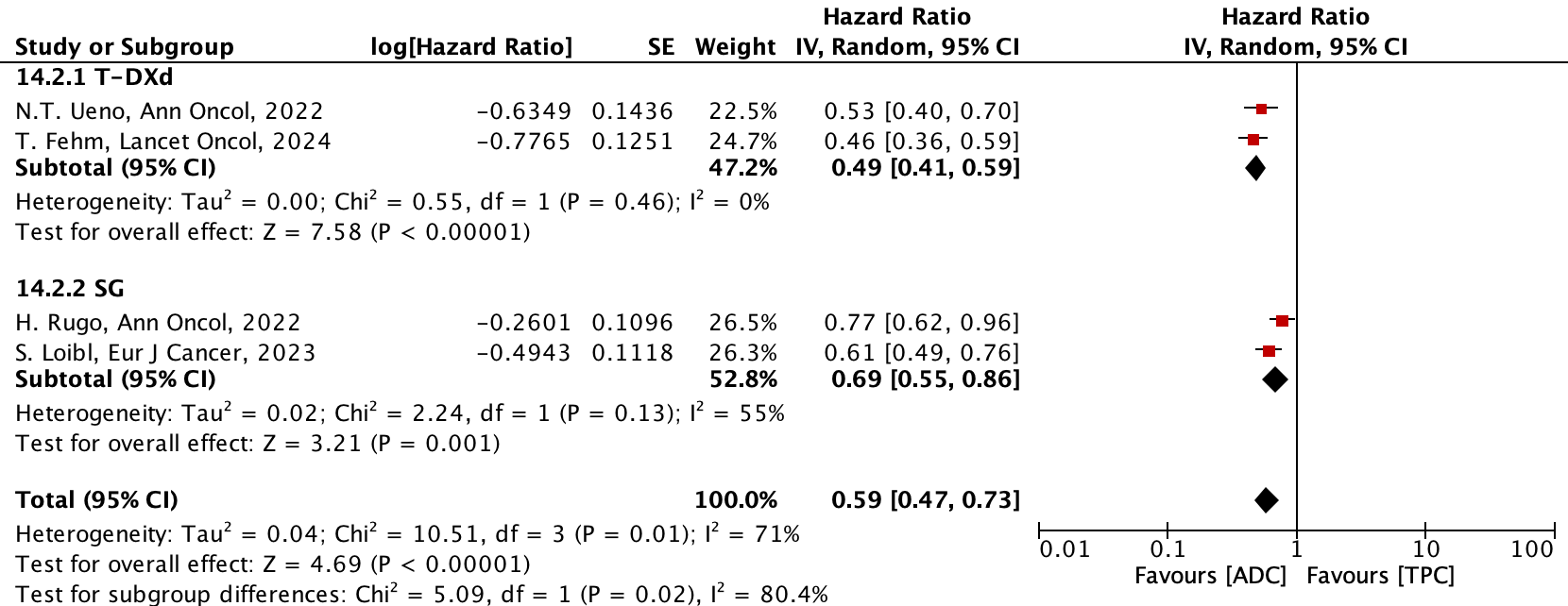


**Figure S14.3 Forest plot of HR of TTD according to EORTC QLQ-C30, outcome: emotional functioning.**

**
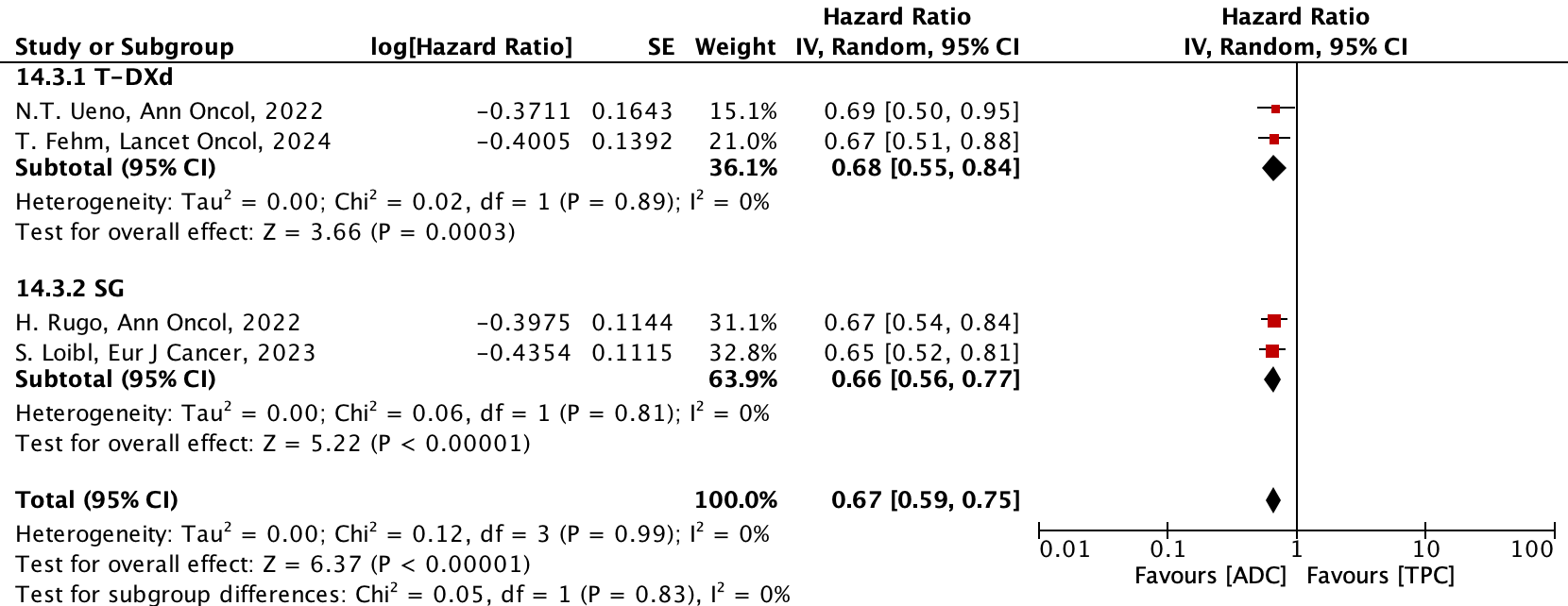
**

**Figure S14.4 Forest plot of HR of TTD according to EORTC QLQ-C30, outcome: social functioning.**


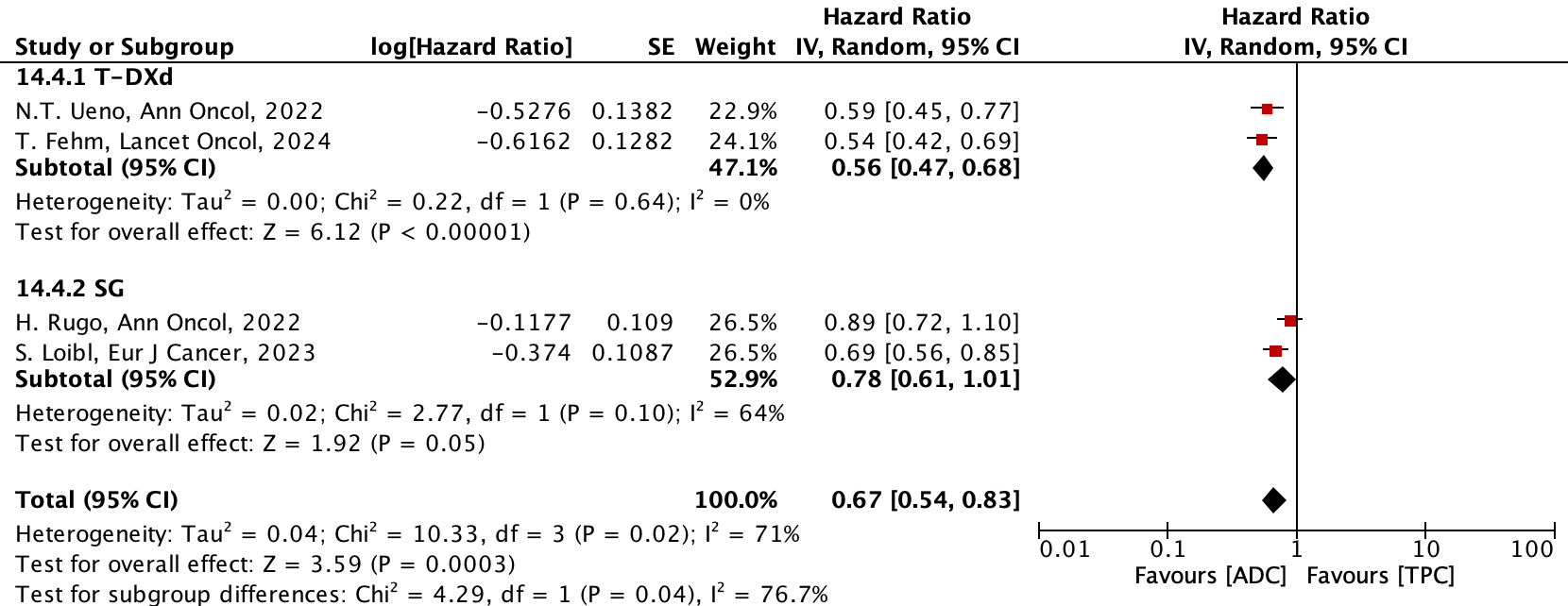


**Figure S14.5 Forest plot of HR of TTD according to EORTC QLQ-C30, outcome: pain symptoms.**

**
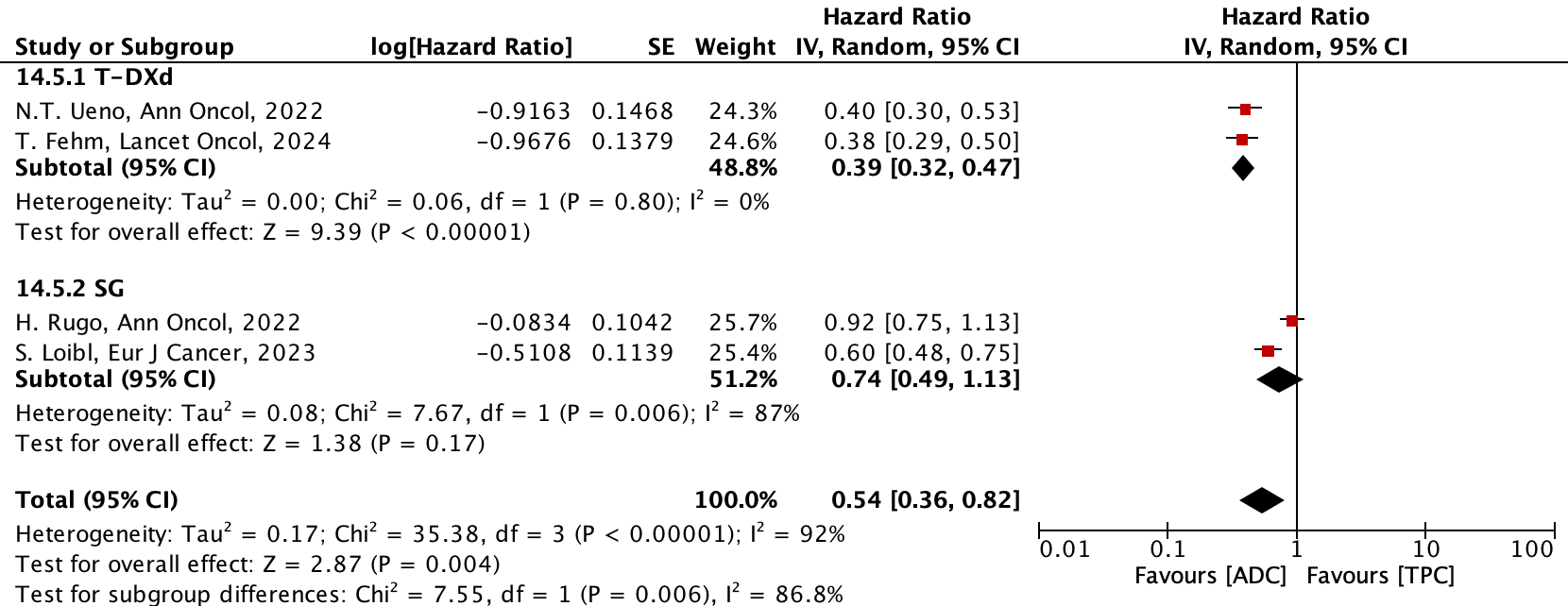
**

**Figure S14.6 Forest plot of HR of TTD according to EORTC QLQ-C30, outcome: fatigue.**

**
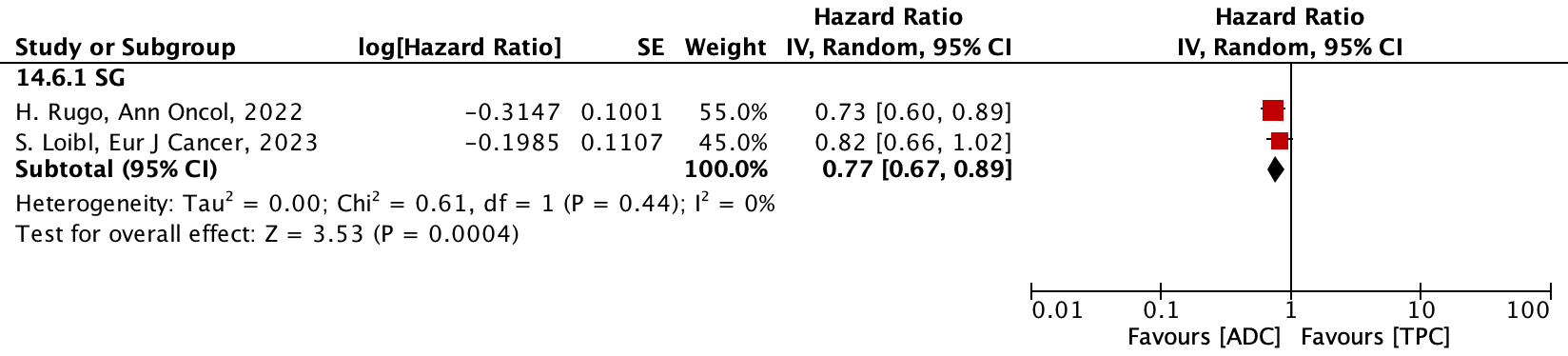
**

**Figure S14.7 Forest plot of HR of TTD according to EORTC QLQ-C30, outcome: nausea and vomiting symptoms.**

**
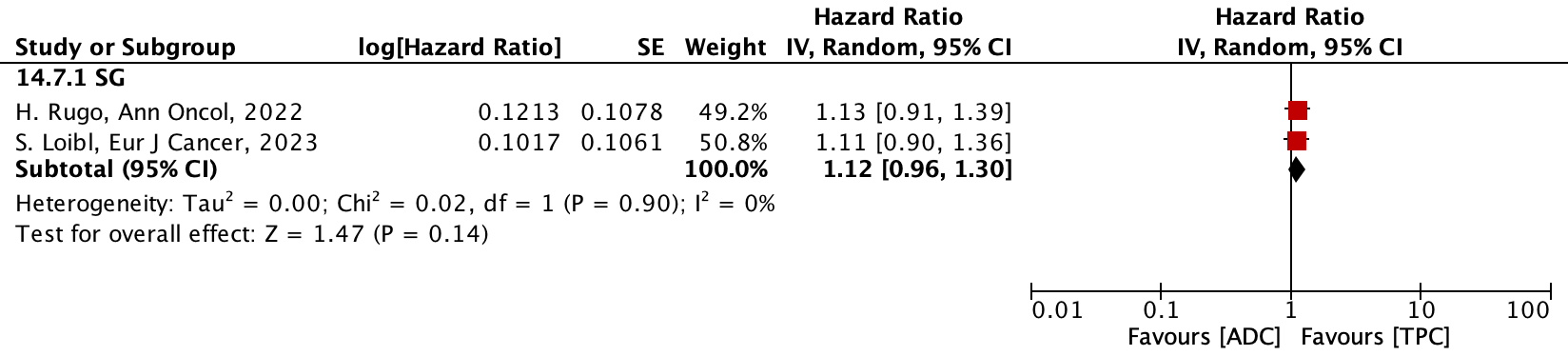
**
